# Angiotensin converting enzyme inhibitors and incidence of lung cancer in a population based cohort of common data model in Korea

**DOI:** 10.1038/s41598-021-97989-8

**Published:** 2021-09-17

**Authors:** Seung-Hwa Lee, Kwang Jin Chun, Jungchan Park, Jinseob Kim, Ji Dong Sung, Rae Woong Park, Jinwook Choi, Kwangmo Yang

**Affiliations:** 1 Rehabilitation & Prevention Center, Heart Vascular Stroke Institute, Samsung Medical Center, Sungkyunkwan University School of Medicine, Seoul, Korea; 2 Department of Biomedical Engineering, Seoul National University College of Medicine, Seoul, Korea; 3 Division of Cardiology, Department of Internal Medicine, Kangwon National University Hospital, College of Medicine, Kangwon National University, Chuncheon, Korea; 4 Department of Anesthesiology and Pain Medicine, Samsung Medical Center, Sungkyunkwan University School of Medicine, Seoul, Korea; 5 Department of Epidemiology, School of Public Health, Seoul National University, Seoul, Korea; 6 Department of Biomedical Sciences, Ajou University Graduate School of Medicine, Suwon, Korea; 7 Center for Health Promotion, Samsung Medical Center, Sungkyunkwan University School of Medicine, 81 Irwon-ro, Gangnam-gu, Seoul, 06351 Korea

**Keywords:** Cardiology, Outcomes research

## Abstract

Contradictory findings exist about association of angiotensin-converting enzyme inhibitor (ACEi) and angiotensin receptor blocker (ARB) with lung cancer development. This was a retrospective observational cohort study that used data from 7 hospitals in Korea, converted to the Observational Medical Outcomes Partnership Common Data Model. The primary outcome was occurrence of lung cancer. A total of 207,794 patients across the 7 databases was included in the final analysis; 33,230 (16%) were prescribed ACEi and 174,564 (84%) were prescribed ARB. Crude analysis adjusted for sex and age showed higher incidence of lung cancer in the ACEi group compared to the ARB group (hazard ratio [HR], 1.46; 95% confidence rate [CI], 1.08–1.97). After propensity-score matching, 30,445 pairs were generated, and there was no difference in incidence of lung cancer between the two groups (HR, 0.93; 95% CI, 0.64–1.35). Patients prescribed ACEi showed no difference in incidence of lung cancer development compared to those using ARB. This finding provides evidence on the association between ACEi and occurrence of lung cancer.

## Introduction

Numerous studies have shown that the Renin–Angiotensin–Aldosterone system (RAAS) affects cancer development^1–3^. The RAAS is composed of different receptors and effectors, such as angiotensin type 1 and 2 receptors, pro-renin receptor, and Angiotensin 2, which are associated with angiogenesis and cell proliferation. Several experimental models have shown that antagonism of the RAAS inhibits tumor growth, angiogenesis, and metastasis^4^. Hence, RAAS is posited to be involved in various cancers, though the findings are controversial and include conflicting data^1,5^.

Several retrospective studies have demonstrated that patients taking the RAAS modifier angiotensin-converting enzyme inhibitor (ACEi) or angiotensin receptor blocker (ARB) had a decreased risk of certain type of cancers^6,7^. However, biological evidence exists for a possible association between ACEIs and increased risk of lung cancer. The use of ACEi causes accumulation of bradykinin in the lung and stimulates the growth of lung cancer^8^. Several cohort studies have reported that patients prescribed ACEi showed increased lung cancer diagnosis compared to those using ARB^8,9^. Generally, ACEis are frequently prescribed in Europe and America, but ARBs are preferred in Asian to avoid the more frequent side effects of ACEi such as cough and angioedema^10^. Hence, the relationship between ACEi and lung cancer may be different across populations. Furthermore, meta-analyses from randomized trials showed conflicting results^1,11^. The association between ACEi and lung cancer development is not clear. The objective of this study was to evaluate the association between ACEi prescription and lung cancer development using de-identified databases from 7 hospitals in Korea.

## Materials and methods

### Data curation

This was a retrospective observational cohort study conducted in accordance with the principles of the Declaration of Helsinki. The Institutional Review Board of Kangwon National University Hospital granted a waiver of approval and informed consent for this study () since we used de-identified data based on longitudinal observational health data from the research network Observational Health Data Sciences and Informatics (OHDSI). The analysis was executed across data from 7 hospitals in Korea (Ajou University Medical Center, Daegu Catholic University Medical Center, Kangwon National University Hospital, Kangdong Sacred Heart Hospital, Pusan National University Hospital, Kyung Hee University Hospital at Gangdong, and Wonkwang University Hospital), and all data were mapped to the Observational Medical Outcome Partnership Common Data Model (OMOP-CDM)^12^. This study followed the Strengthening the Reporting of Observational Studies in Epidemiology (STROBE) reporting guidelines which is provided in the Supplementary Data^13^.

### Cohort definition and outcomes

The target cohort was generated by selecting hypertensive patients on ACEi treatment who were followed up for more than 1 year. For the comparator cohort, we selected patients with ARB prescriptions with the same follow up. Exclusion criteria were age younger than 20 years and diagnosis of lung cancer within 1 year from prescription date of either ACEi or ARB. In addition, we additionally extract cohort of patients on antihypertensive medication other than RAAS inhibitors. We extracted the incidence of each baseline characteristic without an exact number of patients to protect sensitive personal information and maintain a de-identified form of data. The primary outcome was lung cancer occurrence during follow-up.

### Statistical analysis

OHDSI analysis tools are built into the ATLAS interactive analysis platform and the OHDSI Methods Library R packages. OHDSI's open‐source software is publicly available on the GitHub repository (https://github.com/OHDSI/). In addition, concept sets used to define baseline characteristics and study outcomes are available (https://github.com/OHDSI/). ATLAS ver. 2.7.2 was used herein. As OHDSI CDM does not provide exact numbers of patients for each covariate, we presented incidences of baseline characteristics. To minimize the effects of potential confounding factors and selection bias, we underwent propensity score (PS) stratification, used large-scale PS matching, and generated a matched population from the cohorts. Number of covariates used in PS matching was more than 400 according to each cohort, and details are presented at https://feedernet.com/project/103/analysis. Propensity scores were calculated in each database independently, based on available demographic characteristics of each database. Incidence rates were determined per 1000 person-years by dividing the number of lung cancer cases by the total number of person years at risk and multiplying the results by 1000. Cox regression analysis was used to compare cumulative incidence according to RAAS modifier use. Kaplan–Meier estimates were used to construct survival curves after PS matching and were compared with the log-rank test. We then performed random-effects meta-analysis to calculate summary hazard ratio (HR) pooling effect estimates across the databases^14^. We additionally underwent sensitivity analysis with the patients who received RAAS inhibitor at least twice a year. All tests were two-tailed, and p < 0.05 was considered statistically significant.

## Results

**Figure 1:**
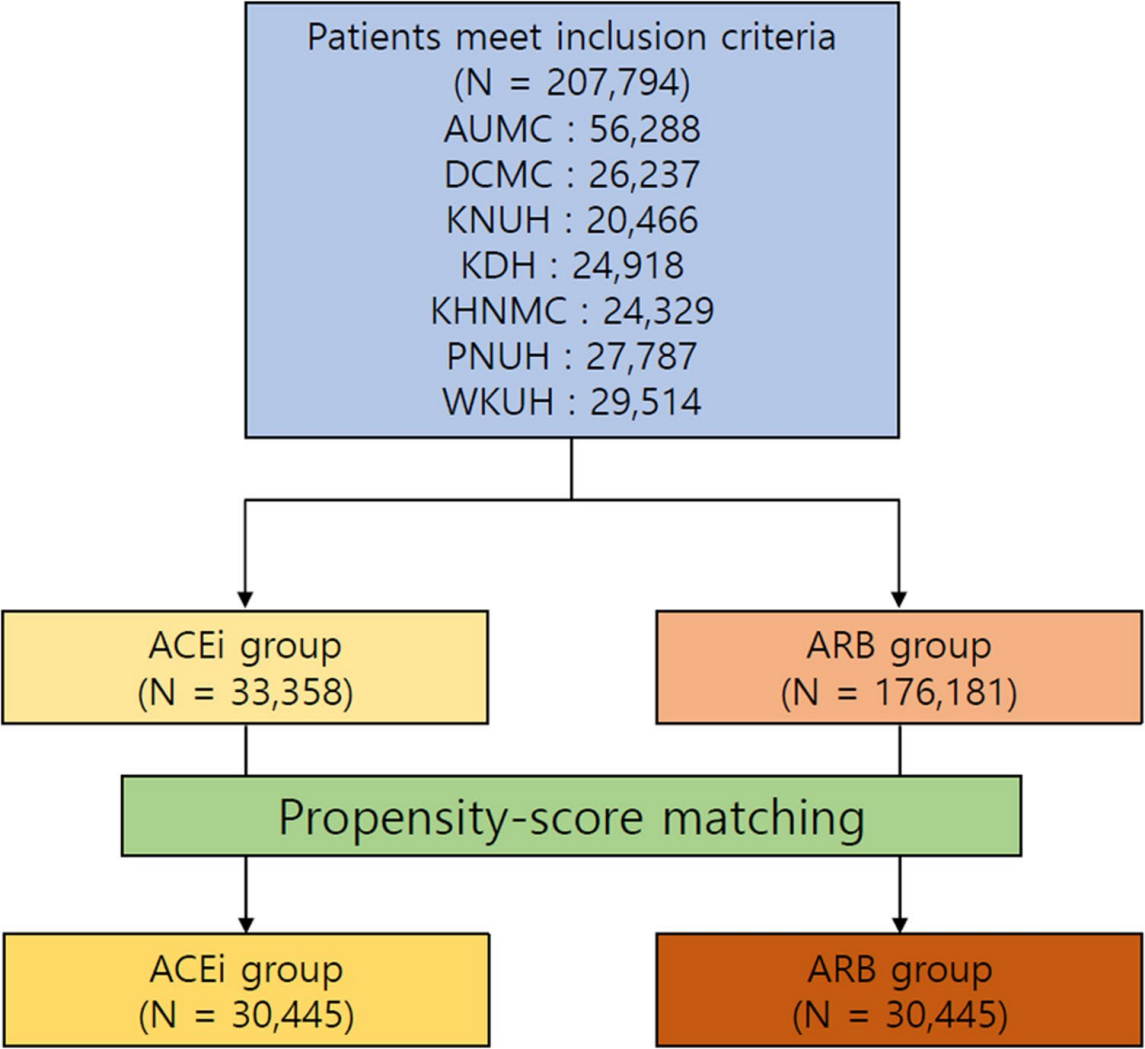
Flowchart of the study.

**Figure 2:**
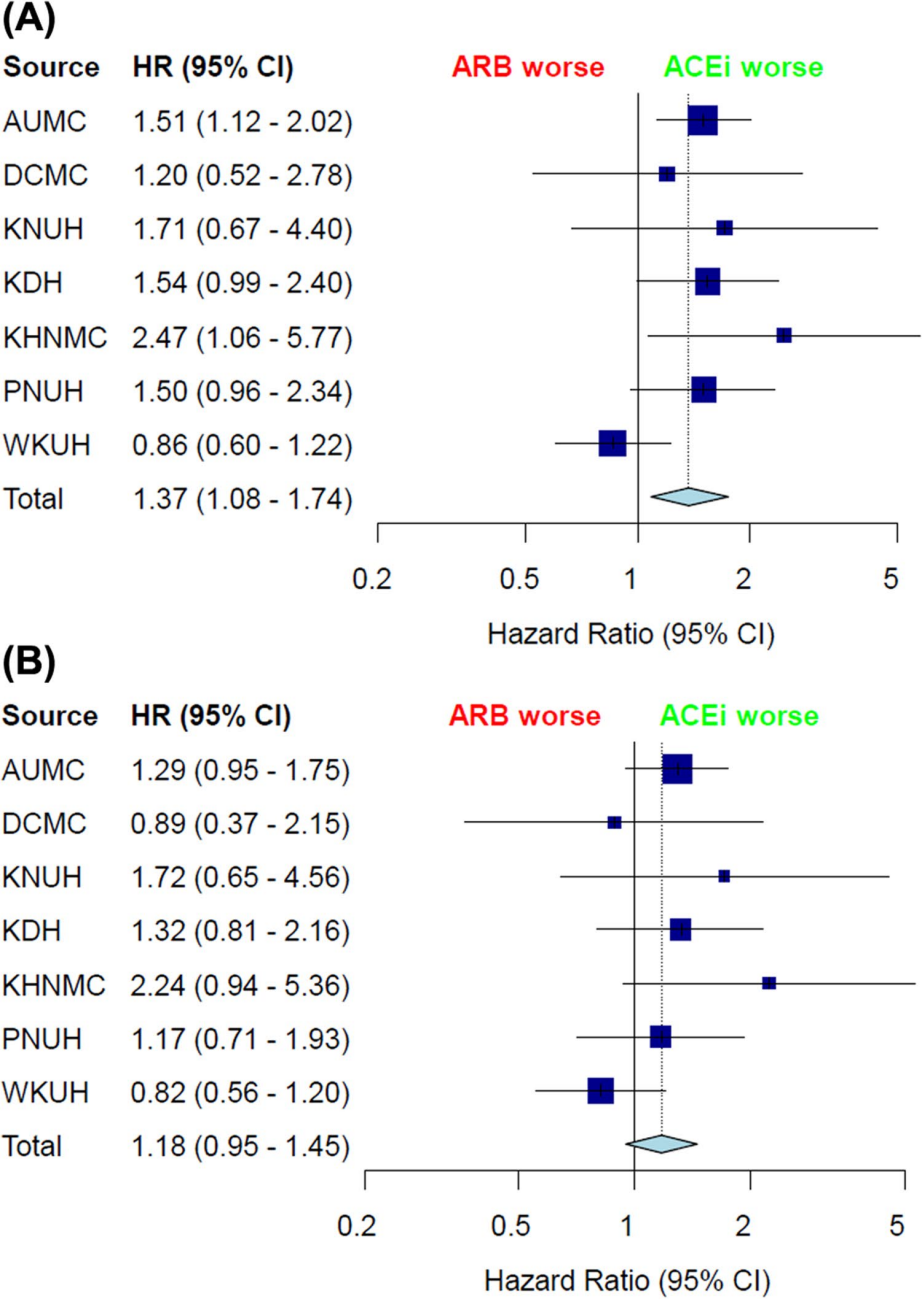
Incidence of lung cancer in the whole cohort (**A**) adjusted for sex and age and (**B**) PS stratified. *AUMC* Ajou University Medical Center, *DCMC* Daegu Catholic University Medical Center, *KNUH* Kangwon National University Hospital, *KDH* Kangdong Sacred Heart Hospital, *KHNMC* Kyung Hee University Hospital at Gangdong, *PNUH* Pusan National University Hospital, *WKUH* Wonkwang University Hospital, *TE* estimated effect, *seTE* standard error of individual studies, *HR* hazard ratio, *CI* confidence interval, *ACEi* angiotensin-converting enzyme inhibitor, *ARB* angiotensin receptor blocker.

**Figure 3:**
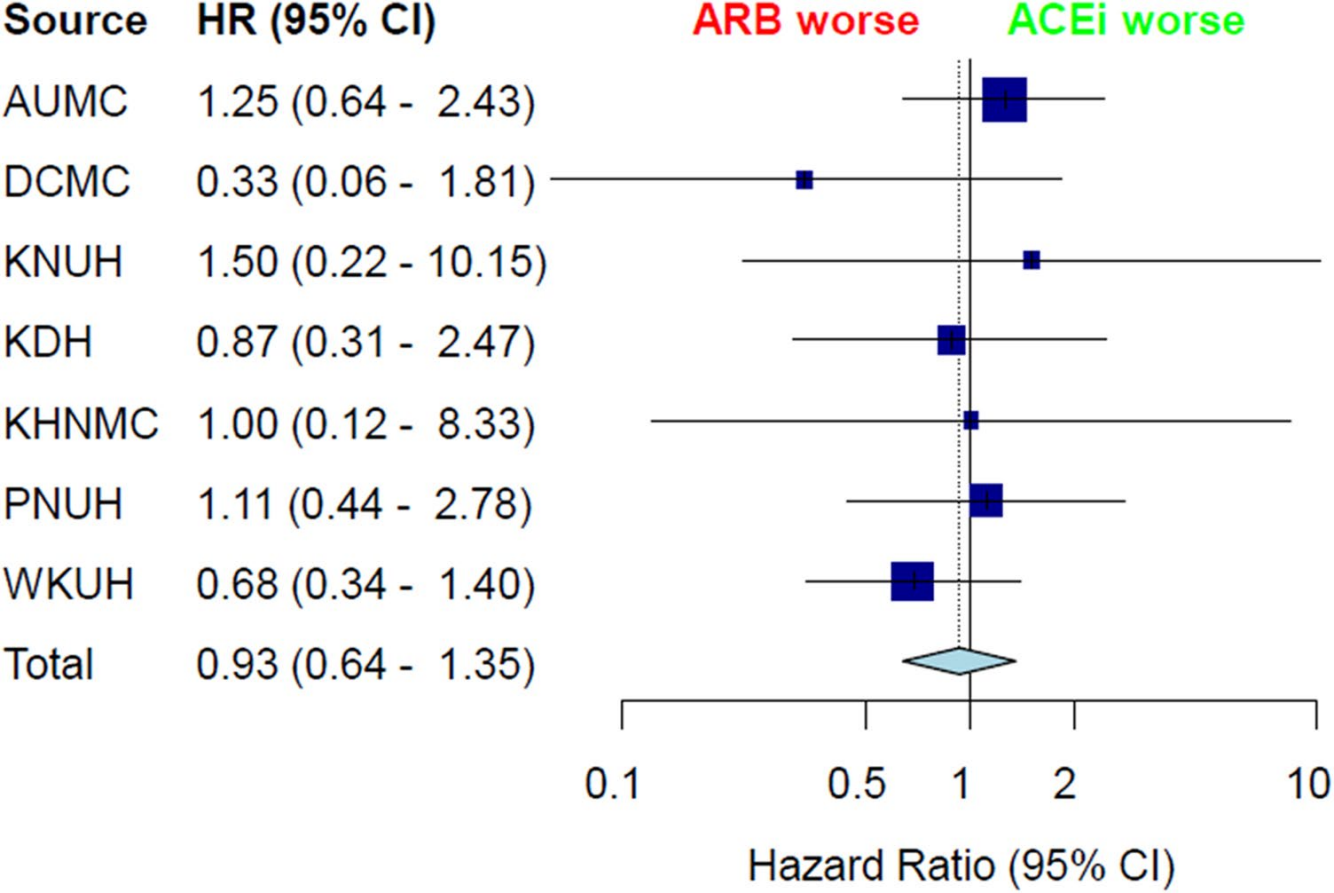
Incidence of lung cancer in the propensity-score matched population. *AUMC* Ajou University Medical Center, *DCMC* Daegu Catholic University Medical Center, *KNUH* Kangwon National University Hospital, *KDH* Kangdong Sacred Heart Hospital, *KHNMC* Kyung Hee University Hospital at Gangdong, *PNUH* Pusan National University Hospital, *WKUH* Wonkwang University Hospital, *TE* estimated effect, *seTE* standard error of individual studies, *HR* hazard ratio, *CI* confidence interval, *ACEi* angiotensin-converting enzyme inhibitor, *ARB* angiotensin receptor blocker.

A total of. 207,794 patients across the 7 hospitals was included in the final analysis. Among these patients, the target cohort comprised 33,230 patients prescribed ACEi, and the comparator cohort was 174,564 patients prescribed ARB in the same time frame (Fig. 1). Attritions of each database are described in Supplementary Figure S1. Baseline characteristics of whole cohort are shown in Table 1. In addition, baseline characteristics of each database are described in Supplementary Table 1–7. Total person years was 124,807 years in the ACEi group and 641,798 years in the ARB group. Incidence of lung cancer was 1.33 per 1000 person years in the ACEi group and 0.96 in the ARB group. Incidence of lung cancer according to database is described in Table 2. Meta-analysis of the whole cohort showed higher incidence of lung cancer in the ACEi group (HR, 1.37; 95% confidence interval [CI], 1.08–1.74) (Fig. 2). After PS-stratification, this difference was not observed (HR, 1.18; 95% CI, 0.95–1.45) (Fig. 2). After PS matching, a total of 30,445 pairs was generated and well balanced (Table 1, Supplementary Fig. S2). Total person years was 100,585 years in the ACEi group and 120,662 years in the ARB group. The incidence of lung cancer was 1.45 per 1000 person years in the ACEi group and 1.14 in the ARB group. Incidence of lung cancer according to database in the PS matched cohort are described in Table 3. In the meta-analysis, no significant difference of lung cancer incidence was found (HR, 0.93; 95% CI, 0.64–1.35) (Fig. 3). Sensitivity analysis with the patients who received RAAS inhibitor at least twice a year, showed the same results. Before adjustment, increased lung cancer incidence was found in the ACEi group. But, adjustment with PS stratification changed this association to the marginal level, and in the PS matched population, the difference of lung cancer incidence was diminished (Supplementary Fig. S3.).

**Table 1:** Baseline characteristics of whole cohort.

	**ACEi group (N = 33,358)**	**ARB group (N = 176,181)**	**SMD**	**Propensity-score matched population**		**SMD**
				**ACEi group (N = 30,445)**	**ARB group (N = 30,445)**	
**Age group**						
25–29	0.39	0.33	0.01	0.4	0.37	0
30–34	0.62	0.69	− 0.01	0.66	0.62	0.01
35–39	1.12	1.35	− 0.02	1.16	1.06	0.01
40–44	1.93	2.37	− 0.03	1.98	1.75	0.02
45–49	3.4	3.63	− 0.01	3.4	3.24	0.01
50–54	4.59	5.13	− 0.03	4.58	4.49	0
55–59	5.75	6.07	− 0.01	5.75	5.72	0
60–64	6.28	6.57	− 0.01	6.23	6.52	− 0.01
65–69	7.08	6.6	0.02	7.03	7.17	− 0.01
70–74	6.68	6.59	0	6.71	6.91	− 0.01
75–79	5.77	5.51	0.01	5.77	5.89	− 0.01
80–84	3.69	3.21	0.03	3.7	3.69	0
85–89	1.73	1.26	0.04	1.66	1.67	0
Female	20.25	24.16	− 0.09	21.01	21.21	0
**Medical history: general**						
Acute respiratory disease	1.54	1.16	0.03	1.45	1.42	0
Dementia	0.88	1.31	− 0.04	0.89	0.79	0.01
Depressive disorder	0.97	1.38	− 0.04	1.01	0.98	0
Diabetes mellitus	10.88	10.31	0.02	10.9	10.96	0
Gastroesophageal reflux disease	1.57	1.76	− 0.02	1.57	1.51	0
Gastrointestinal hemorrhage	0.95	0.7	0.03	0.89	0.87	0
Hyperlipidemia	7.61	8.32	− 0.03	7.73	7.23	0.02
Lesion of liver	1.42	1.32	0.01	1.4	1.28	0.01
Obesity	0.11	0.31	− 0.04	0.11	0.12	0
Osteoarthritis	0.56	0.88	− 0.04	0.6	0.54	0.01
Pneumonia	2.32	1.64	0.05	2.22	2.29	0
Renal impairment	2.84	3.43	− 0.03	2.91	2.83	0
Urinary tract infectious disease	0.81	0.66	0.02	0.8	0.8	0
Visual system disorder	4.22	4.65	− 0.02	4.25	4.25	0
**Medical history: Cardiovascular disease**						
Atrial fibrillation	2.18	1.57	0.04	2.28	2.41	− 0.01
Coronary arteriosclerosis	3.88	1.79	0.13	3.81	3.92	− 0.01
Heart disease	29.56	13.85	0.39	27.91	28.88	− 0.02
Heart failure	7	2.36	0.22	6.34	6.48	− 0.01
Ischemic heart disease	19.56	6.51	0.4	17.88	18.34	− 0.01
Peripheral vascular disease	2.81	3.29	− 0.03	2.81	2.79	0
**Medical history: Neoplasms**						
Malignant neoplasm of anorectum	0.27	0.23	0.01	0.26	0.22	0.01
Malignant neoplastic disease	3.77	4.04	− 0.01	3.57	3.39	0.01
Malignant tumor of breast	0.27	0.3	− 0.01	0.27	0.24	0.01
Primary malignant neoplasm of prostate	0.27	0.25	0	0.26	0.28	0

Data are presented as %.

*ACEi* Angiotensin converting enzyme inhibitoir, *ARB* Angiotensin receptor blocker, *SMD* standardized mean difference.

Current table showed the aggregated balance before and after matching only for limited covariates from 7 databases.

**Table 2:** Incidence of lung cancer in the whole cohort.

Hospitals	ACEi group	ARB group	Lung cancer in ACEi group*	Lung cancer in ARB group*	Adjusted HR(95% CI)	p-value	PS-stratification	
							Adjusted HR (95% CI)	p-value
AUMC	7355	48,933	2.00 (1.51–2.57)	1.27 (1.12–1.44)	1.51 (1.11–2.00)	0.006	1.29 (0.95–1.74)	0.097
DCMC	7011	19,226	0.33 (0.15–0.61)	0.28 (0.17–0.42)	1.20 (0.50–2.65)	0.671	0.89 (0.35–2.07)	0.792
KNUH	1346	19,120	1.00 (0.36–2.15)	0.58 (0.43–0.77)	1.71 (0.59–3.92)	0.265	1.72 (0.58–4.10)	0.278
KDH	5335	19,583	1.36 (0.91–1.93)	0.88 (0.68–1.10)	1.54 (0.98–2.37)	0.056	1.32 (0.80–2.13)	0.272
KHNMC	736	23,593	2.42 (0.96–4.90)	0.98 (0.77–1.21)	2.47 (0.96–5.22)	0.036	2.24 (0.85–4.88)	0.069
PNUH	4654	23,133	1.87 (1.23–2.68)	1.25 (0.99–1.54)	1.50 (0.94–2.30)	0.076	1.17 (0.71–1.91)	0.531
WKUH	6921	22,593	1.29 (0.93–1.72)	1.37 (1.14–1.63)	0.86 (0.59–1.21)	0.395	0.82 (0.56–1.19)	0.31

*Incidence rate are presented as per 1000 years (95% confidential interval).

*ACEi* Angiotension converting enzyme inhibitor, *ARB* Angiotension receptor blocker, *PS* propensity-score, *AUMC* Ajou University Medical Center, *DCMC* Daegu Catholic University Medical Center, *KNUH* Kangwon National University Hospital, *KDH* Kangdong Sacred Heart Hospital, *KHNMC* Kyung Hee University Hospital at Gangdong, *PNUH* Pusan National University Hospital, *WKUH* Wonkwang University Hospital, *HR* hazard ratio, *CI* confidence interval.

**Table 3:** Incidence of lung cancer in the propensity-score matched population.

Hospitals	ACEi group	ARB group	Lung cancer in ACEi group*	Lung cancer in ARB group*	Adjusted HR (95% CI)	p-value
AUMC	7026	7026	1.97 (1.49–2.55)	1.62 (1.21–2.11)	1.25 (0.65–2.45)	0.51
DCMC	6380	6380	1.10 (0.47–2.12)	0.59 (0.32–0.96)	0.33 (0.05–1.45)	0.204
KNUH	1335	1335	1.01 (0.36–2.19)	0.92 (0.33–1.98)	1.50 (0.25–11.39)	0.678
KDH	4457	4457	1.30 (0.83–1.90)	0.88 (0.53–1.37)	0.88 (0.31–2.44)	0.801
KHNMC	734	734	2.43 (0.97–4.92)	0.82 (0.14–2.52)	1.00 (0.12–8.33)	0.99
PNUH	4474	4474	1.71 (0.11–2.51)	1.35 (0.82–2.07)	1.11 (0.45–2.80)	0.822
WKUH	6039	6039	1.04 (0.71–1.46)	1.26 (0.89–1.73)	0.68 (0.33–1.37)	0.297

*Incidence rate are presented as per 1000 years (95% confidential interval).

*ACEi* Angiotension converting enzyme inhibitor, *ARB* Angiotension receptor blocker, *PS* propensity-score, *AUMC* Ajou University Medical Center, *DCMC* Daegu Catholic University Medical Center, *KNUH* Kangwon National University Hospital, *KDH* Kangdong Sacred Heart Hospital, *KHNMC* Kyung Hee University Hospital at Gangdong.

We additionally compare the incidence of lung cancer between either ACEi or ARB and antihypertensive agent other than RAAS inhibitor, such as calcium channel blockers, diuretics, or beta-blockers. Baseline characteristics of ACEi and antihypertensive agent other than RAAS inhibitor group before and after PS matching were described in Supplementary table S8. Supplementary table S9, S10 and supplementary figure S4 showed the incidence of lung cancer in the ACEi and antihypertensive agent other than RAAS inhibitor group before and after propensity-score adjustment. After PS matching, ACEi group showed lower incidence of lung cancer compared to other hypertensive agent group (HR, 0.60; 95% CI, 0.41–0.90). Supplementary table S11-13 and supplementary figure S5 showed baseline characteristics and incidence of lung cancer between ARB and other antihypertensive groups. After PS matching, the incidence of lung cancer showed no difference between two groups (HR, 1.00; 95% CI, 0.67–1.50).

## Discussion

In the current study, hypertensive patients prescribed ACEi did not appear to be associated with a greater incidence of lung cancer compared with patients using ARB. The results of our study add evidence to the conflicting results about effect of ACEi prescription on lung cancer development.

ACE is the main bradykinin-inactivating metallopeptidase in humans, and ACEi induces accumulation of bradykinin in the lung and may stimulate growth of lung cancer^8,15^. As angiotensin II has an important role in development, progression, and dissemination of cancer by stimulating cell proliferation, angiogenesis, and inflammation, RAAS modulator may have a protective role against cancer occurence^7^. Furthermore, addional analysis of current study presented that ACEi showed decreased incidence of lung cancer compared to antihypertensive agent other than RAAS inhibitor group, but it needs further evaluation. Conflicting results exist about the relationship between ACEi and lung cancer. Several cohort studies reported that patients prescribed ACEi showed higher lung cancer incidence than patients prescribed ARB^8,9^. Contrarily, meta-analysis of randomized trials showed that incidence of lung cancer was similar or higher in patients prescribed ARB compared to a control group^1,16^. Another issue in study of ARB and cancer is that some sartan products appeared to have been contaminated with N-nitrodosimethylamine and N-nitrosodiethylamine, which are probably carcinogenic to human^17^. More data are needed to evaluate the association between ACEi and lung cancer.

The results of the current study showed that incidence of lung cancer was similar between ACEi and ARB prescription, and this correlate well with a prior meta-analysis. There are several explanations about this. First, we used large datasets based on OMOP-CDM and performed PS matching using a large-scale propensity score algorithm. Using a larger covariate set and including all covariates in the PS model produces improved performance^18^, in agreement with previous randomized trials. Second, our study used databases from Korea, in which the National Health Screening Program is one of the world’s largest health screening programs aimed for early detection for cancer^18^. Due to the nationwide free cancer screening in this program, the screening rate is relatively high in Korea^19^, and even mild symptoms such as cough, a frequent side effect of ACEi, could lead to a differential diagnosis using x-ray or chest computed tomography. Therefore, patients prescribed ACEi might have had a greater chance of being evaluated for a lung lesion, and this may have led to higher incidence of lung cancer. Third, prescription ratio of ARB may affect the result. Contrary to Western countries, ARBs are almost 20 times more frequently prescribed to hypertensive patients than are ACEis because of frequent side effects^19^. Previous cohort study in the United Kingdom showed that ACEi prescription rate was 10 times higher than that of ARB, and this might have affected the conflicting results of the present study^8^.

The strength of our study is that we used real-world data based on Electronic Health Records (EHR) of hospitals. Most large, real-world databases were based on health insurance claims, which have disadvantages of lack of clinical data, medical histories, and therapeutic courses. Therefore, EHR-based clinical data provide more objective results than do claims-based data^20^. The results of our study are in agreement with prior meta-analyses of randomized studies and provide evidence from real-world data-based studies in agreement with prior published CDM-based studies^21,22^.

The results of this study should be interpreted with consideration of the following limitations. First, this was a retrospective study. Despite our efforts to adjust all confounding factors by large-scale PS matching analysis, some of the covariates were not well balanced in the propensity score matched population, and unmeasured factors might have affected the results. In addition, reason for prescription either ACEi or ARB could not be evaluated. Second, there is lack information on dose and duration because of limitations of the CDM databases. Third, the study does not provide a balance between risk of lung cancer and benefit of life expectancy for ACEi treatment. Despite these limitations, this study provides the first real-world evidence from CDM extracted from EHR-based datasets and valuable information about association between ACEi treatment and lung cancer.

## Conclusion

Patients prescribed ACEi showed no difference in incidence of lung cancer development compared to those using ARB. This finding is additional evidence of the association between ACEi and occurrence of lung cancer.

## Supplementary Information

Supplementary Information 1.

Supplementary Information 2.

Supplementary Information 3.

Supplementary Information 4.

Supplementary Information 5.

Supplementary Information 6.

Supplementary Information 7.
