# Supplementary figures and images for: Angiotensin converting enzyme inhibitors and incidence of lung cancer in a population based cohort of common data model in Korea
Source: Sci Rep. 2021 Sep 17;11:18576. doi: 10.1038/s41598-021-97989-8 (PMC8448874; doi:10.1038/s41598-021-97989-8)

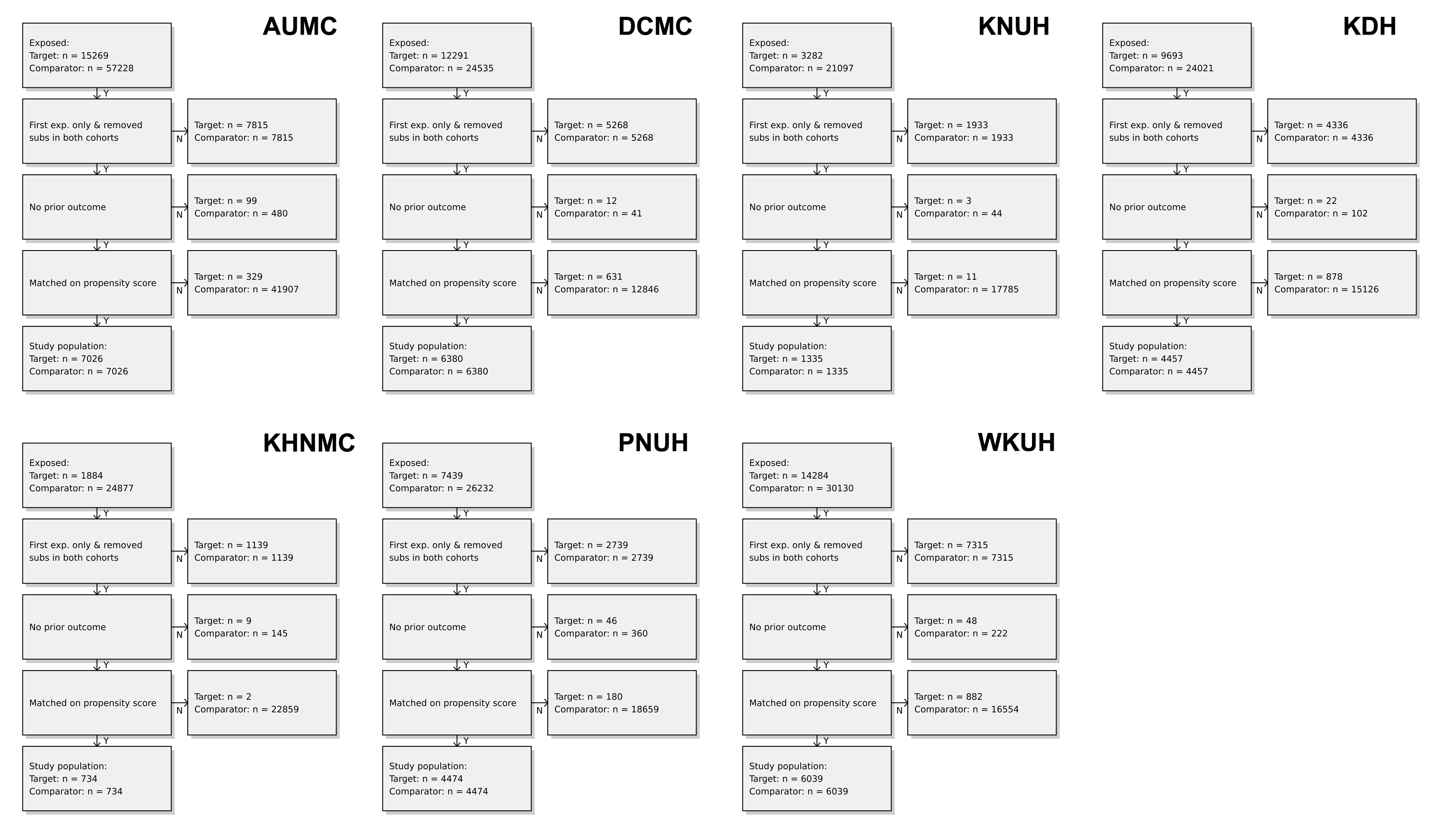

Supplement: Supplementary file 1 — Supplementary Information 1. [file 41598_2021_97989_MOESM1_ESM.tif]

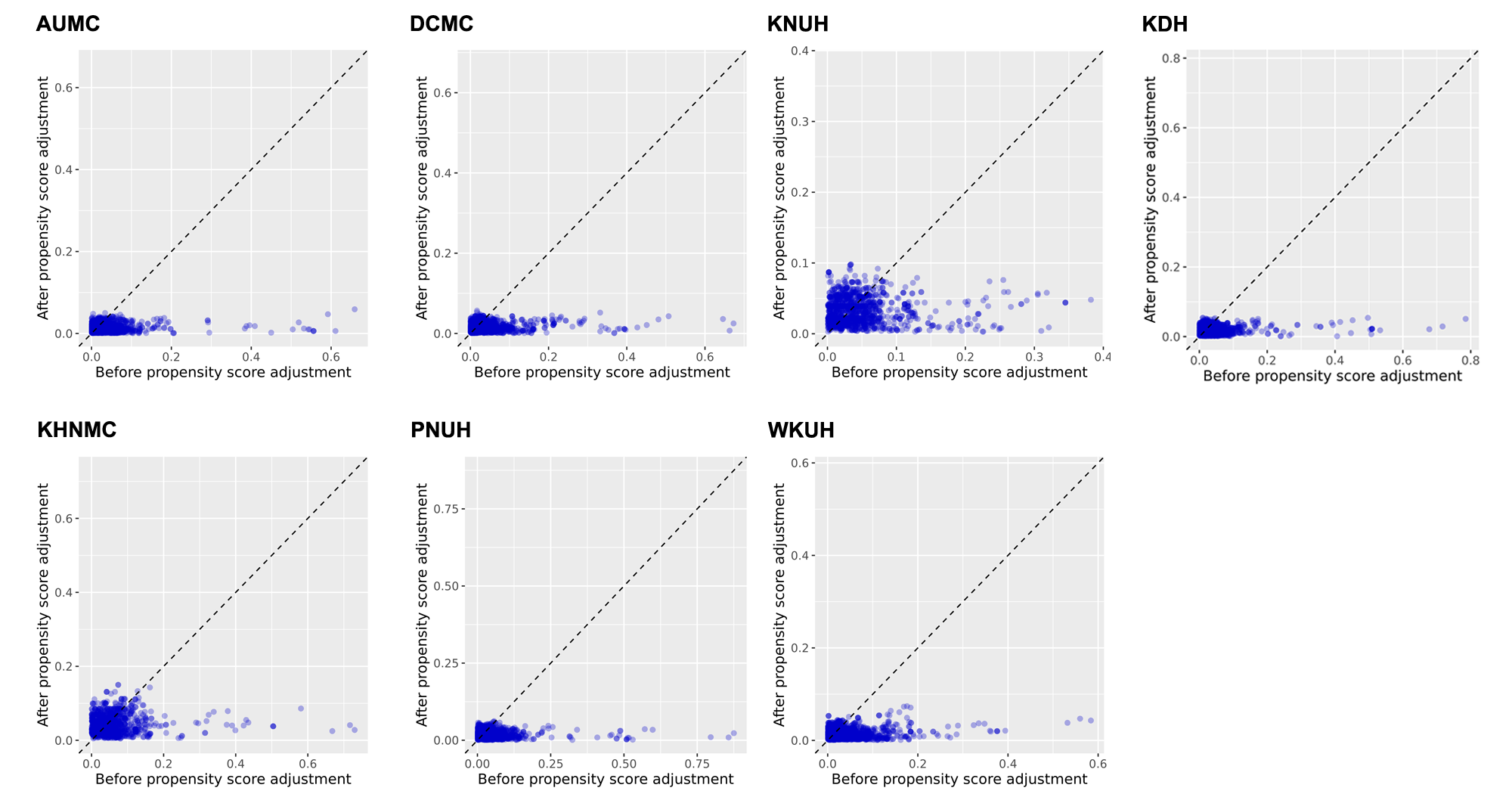

Supplement: Supplementary file 2 — Supplementary Information 2. [file 41598_2021_97989_MOESM2_ESM.tif]

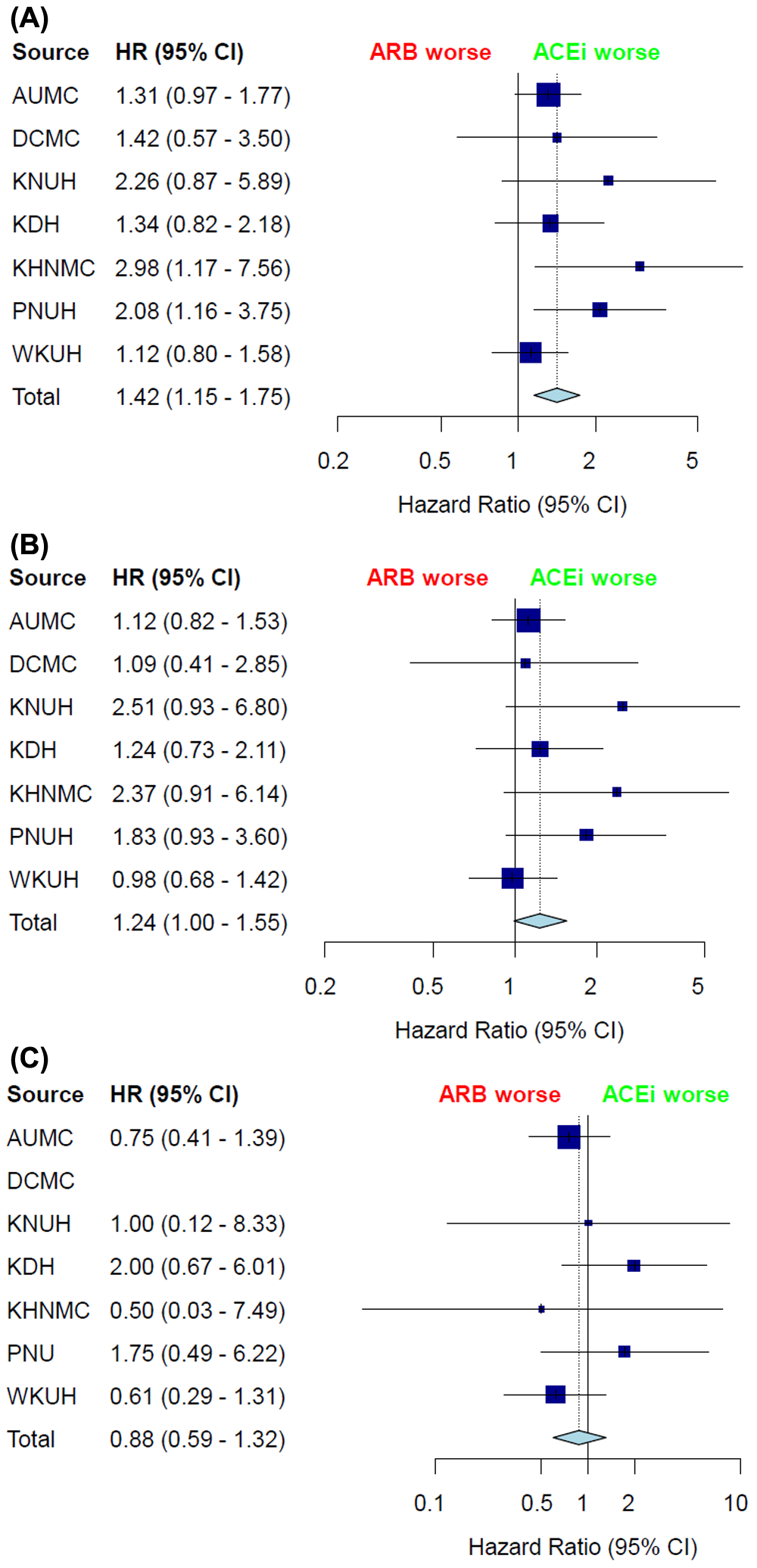

Supplement: Supplementary file 3 — Supplementary Information 3. [file 41598_2021_97989_MOESM3_ESM.tif]

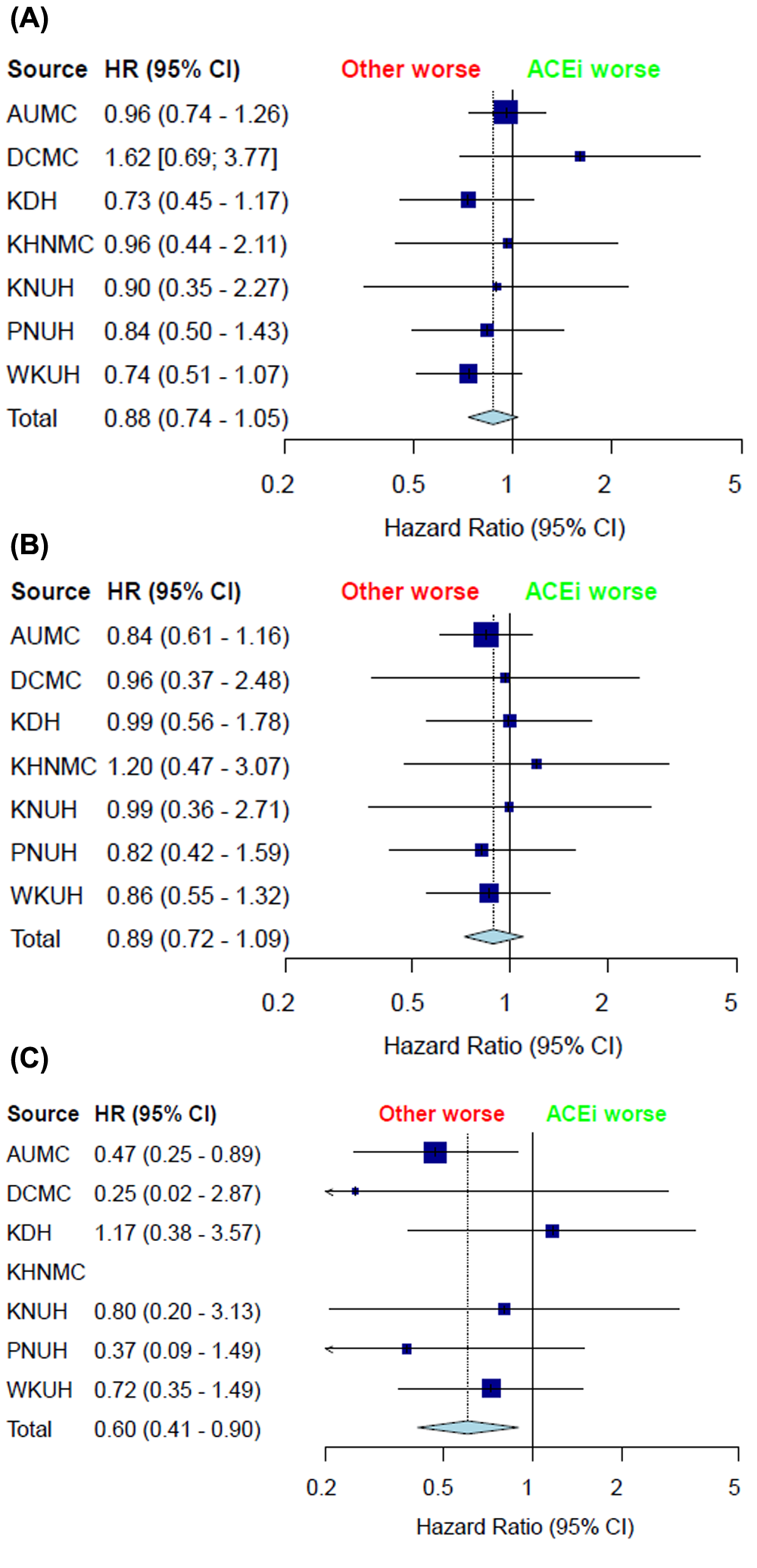

Supplement: Supplementary file 4 — Supplementary Information 4. [file 41598_2021_97989_MOESM4_ESM.tif]

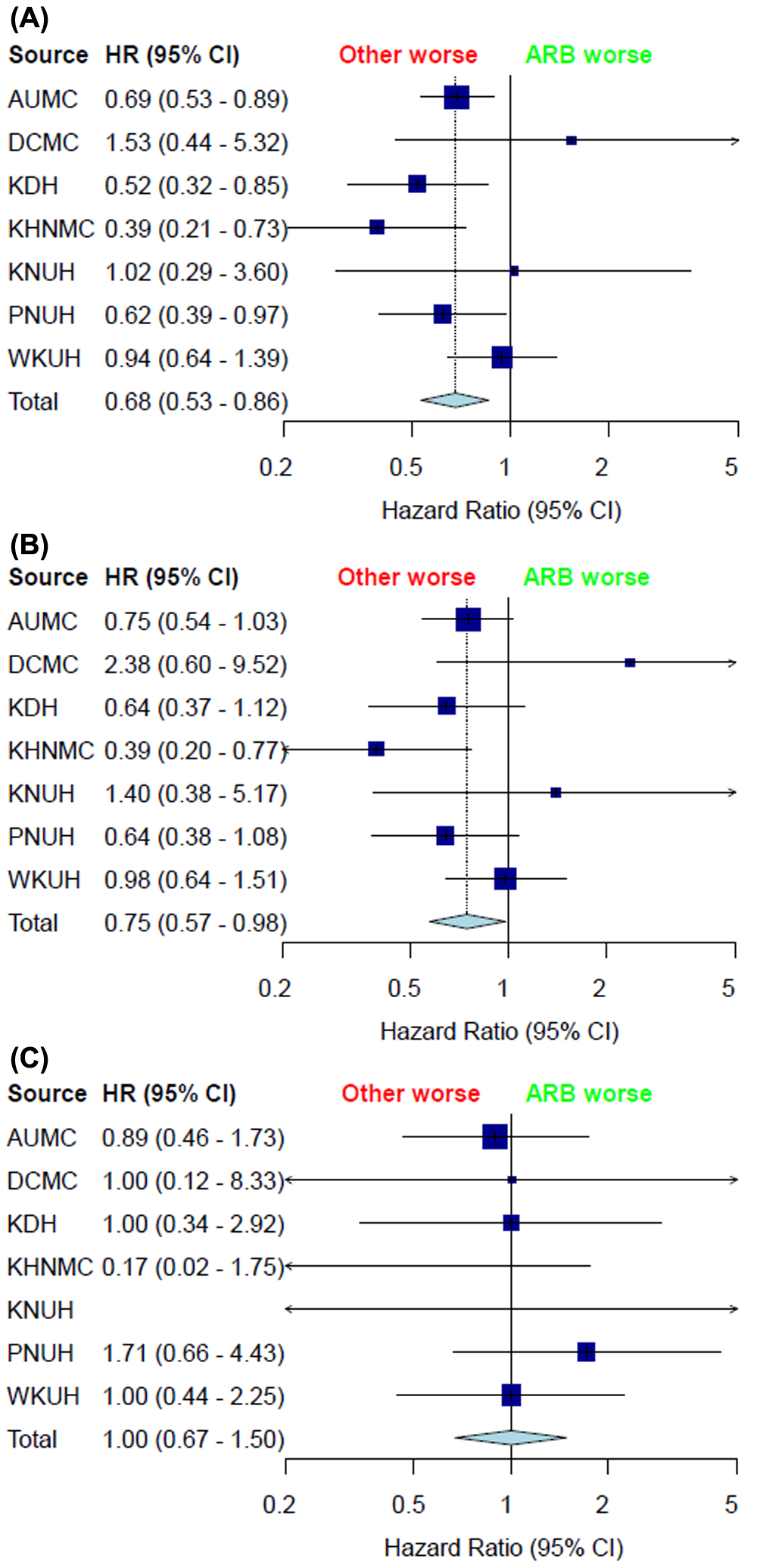

Supplement: Supplementary file 5 — Supplementary Information 5. [file 41598_2021_97989_MOESM5_ESM.tif]
