# Supplementary material for: Angiotensin converting enzyme inhibitors and incidence of lung cancer in a population based cohort of common data model in Korea
Source: Sci Rep. 2021 Sep 17;11:18576. doi: 10.1038/s41598-021-97989-8 (PMC8448874; doi:10.1038/s41598-021-97989-8)
Supplement: Supplementary file 6 — Supplementary Information 6. [file 41598_2021_97989_MOESM6_ESM.docx]

**Supplementary figure legends**

**Supplementary Figure S1.** Attritions of each database.

Abbreviations: AUMC, Ajou University Medical Center; DCMC, Daegu Catholic University Medical Center; KNUH, Kangwon National University Hospital; KDH, Kangdong Sacred Heart Hospital; KHNMC, Kyung Hee University Hospital at Gangdong; PNUH, Pusan National University Hospital; WKUH, Wonkwang University Hospital

**Supplementary Figure S2.** Before and after propensity score adjustment of each database.

Abbreviations: AUMC, Ajou University Medical Center; DCMC, Daegu Catholic University Medical Center; KNUH, Kangwon National University Hospital; KDH, Kangdong Sacred Heart Hospital; KHNMC, Kyung Hee University Hospital at Gangdong; PNUH, Pusan National University Hospital; WKUH, Wonkwang University Hospital

**Supplementary Figure S3.** Incidence of lung cancer in the cohort with the patients who received RAAS inhibitor at least twice a year (A) adjusted for sex and age, (B) PS stratified and (C) PS matched population.

Abbreviations: AUMC, Ajou University Medical Center; DCMC, Daegu Catholic University Medical Center; KNUH, Kangwon National University Hospital; KDH, Kangdong Sacred Heart Hospital; KHNMC, Kyung Hee University Hospital at Gangdong; PNUH, Pusan National University Hospital; WKUH, Wonkwang University Hospital; TE, estimated effect; seTE, standard error of individual studies; HR, hazard ratio; CI, confidence interval; ACEi, angiotensin-converting enzyme inhibitor; ARB, angiotensin receptor blocker; RAAS, renin-angiotensin-aldosterone system.

**Supplementary Figure S4.** **.** Incidence of lung cancer in the cohort of ACEi and antihypertensive agent other than RAAS inhibitor (A) adjusted for sex and age, (B) PS stratified and (C) PS matched population.

Abbreviations: AUMC, Ajou University Medical Center; DCMC, Daegu Catholic University Medical Center; KNUH, Kangwon National University Hospital; KDH, Kangdong Sacred Heart Hospital; KHNMC, Kyung Hee University Hospital at Gangdong; PNUH, Pusan National University Hospital; WKUH, Wonkwang University Hospital; TE, estimated effect; seTE, standard error of individual studies; HR, hazard ratio; CI, confidence interval; ACEi, angiotensin-converting enzyme inhibitor; RAAS, renin-angiotensin-aldosterone system.

**Supplementary Figure S5** **.** Incidence of lung cancer in the cohort of ARB and antihypertensive agent other than RAAS inhibitor (A) adjusted for sex and age, (B) PS stratified and (C) PS matched population.

Abbreviations: AUMC, Ajou University Medical Center; DCMC, Daegu Catholic University Medical Center; KNUH, Kangwon National University Hospital; KDH, Kangdong Sacred Heart Hospital; KHNMC, Kyung Hee University Hospital at Gangdong; PNUH, Pusan National University Hospital; WKUH, Wonkwang University Hospital; TE, estimated effect; seTE, standard error of individual studies; HR, hazard ratio; CI, confidence interval; ARB, angiotensin receptor blocker; RAAS, renin-angiotensin-aldosterone system.

**Supplementary Table legends**

**Supplementary Table S1.** Baseline characteristics of AUMC database.

**Supplementary Table S2.** Baseline characteristics of DCMC database.

**Supplementary Table S3.** Baseline characteristics of KNUH database.

**Supplementary Table S4.** Baseline characteristics of KDH database.

**Supplementary Table S5.** Baseline characteristics of KHNMC database.

**Supplementary Table S6.** Baseline characteristics of PNUH database

**Supplementary Table S7.** Baseline characteristics of WKUH database.

**Supplementary Table S8.** Baseline characteristics of ACEi group and antihypertensive agent other than RAAS inhibitor.

**Supplementary Table S9**. Incidence of lung cancer in the ACEi and other cohort.

**Supplementary Table S10.** Incidence of lung cancer in the propensity-score matched ACEi and other cohort

**Supplementary Table S11.** Baseline characteristics of ARB group and antihypertensive agent other than RAAS inhibitor.

**Supplementary Table S12**. Incidence of lung cancer in the ARB and other cohort.

**Supplementary Table S13.** Incidence of lung cancer in the propensity-score matched ARB and other cohort
