# Supplementary material for: Angiotensin converting enzyme inhibitors and incidence of lung cancer in a population based cohort of common data model in Korea
Source: Sci Rep. 2021 Sep 17;11:18576. doi: 10.1038/s41598-021-97989-8 (PMC8448874; doi:10.1038/s41598-021-97989-8)
Supplement: Supplementary file 7 — Supplementary Information 7. [file 41598_2021_97989_MOESM7_ESM.docx]

**Supplementary Table 1.** Baseline characteristics of AUMC database.

|  | **ACEi group (N = 7,355)** | **ARB group (N = 8,933)** | **SMD** | **Propensity-score matched population** | | |
| --- | --- | --- | --- | --- | --- | --- |
|  |  |  |  | **ACEi group (N = 7,026)** | **ARB group (N = 7,026)** | **SMD** |
| Age group |  |  |  |  |  |  |
| 20-24 | 1.6 | 0.9 | 0.07 | 1.6 | 1.7 | -0.01 |
| 25-29 | 1.2 | 0.9 | 0.03 | 1.2 | 1.1 | 0.01 |
| 30-34 | 2.3 | 2 | 0.02 | 2.3 | 2.5 | -0.01 |
| 35-39 | 3.6 | 3.9 | -0.02 | 3.7 | 3 | 0.04 |
| 40-44 | 5.6 | 6.9 | -0.05 | 5.8 | 4.8 | 0.04 |
| 45-49 | 9 | 9.9 | -0.03 | 9 | 8.3 | 0.02 |
| 50-54 | 10 | 12.1 | -0.07 | 10.2 | 9.6 | 0.02 |
| 55-59 | 11.2 | 12.1 | -0.03 | 11.3 | 11.4 | 0 |
| 60-64 | 12 | 12.6 | -0.02 | 12 | 12.9 | -0.02 |
| 65-69 | 13.8 | 12.3 | 0.05 | 13.5 | 14.1 | -0.02 |
| 70-74 | 11.4 | 11.3 | 0 | 11.3 | 11.5 | 0 |
| 75-79 | 9 | 8.7 | 0.01 | 8.9 | 9.7 | -0.03 |
| 80-84 | 5.8 | 4.4 | 0.06 | 5.8 | 5.8 | 0 |
| 85-89 | 2.5 | 1.6 | 0.06 | 2.4 | 2.4 | 0 |
| 90-94 | 0.9 | 0.4 | 0.07 | 0.8 | 0.9 | -0.01 |
| Female | 36.2 | 45.9 | -0.2 | 37.3 | 36.8 | 0.01 |
| Medical history: General | |  |  |  |  |  |
| Acute respiratory disease | 2.6 | 1.7 | 0.07 | 2.4 | 2.3 | 0.01 |
| Chronic liver disease | 1.7 | 1.4 | 0.02 | 1.7 | 1.6 | 0 |
| Chronic obstructive lung disease | 3.2 | 1.6 | 0.1 | 2.9 | 3 | 0 |
| Dementia | 1.1 | 1.6 | -0.04 | 1.1 | 1 | 0.01 |
| Depressive disorder | 1.5 | 1.8 | -0.02 | 1.5 | 1.6 | -0.01 |
| Diabetes mellitus | 26.1 | 23.1 | 0.07 | 25.8 | 27.3 | -0.03 |
| Gastroesophageal reflux disease | 2.5 | 3.3 | -0.05 | 2.4 | 2.3 | 0 |
| Gastrointestinal hemorrhage | 1.6 | 1.1 | 0.05 | 1.5 | 1.5 | 0 |
| Hyperlipidemia | 5.4 | 5.7 | -0.02 | 5.6 | 5.4 | 0.01 |
| Lesion of liver | 2.8 | 2.6 | 0.02 | 2.8 | 2.6 | 0.01 |
| Obesity | 0.4 | 1.1 | -0.08 | 0.4 | 0.4 | 0.01 |
| Osteoarthritis | 0.6 | 1.4 | -0.09 | 0.6 | 0.6 | -0.01 |
| Pneumonia | 6 | 2.8 | 0.16 | 5.3 | 5.6 | -0.01 |
| Psoriasis | 0.2 | 0.2 | 0 | 0.2 | 0.2 | -0.01 |
|  | **ACEi group (N = 7,355)** | **ARB group (N = 48,933)** | **SMD** | **Propensity-score matched population** | | |
|  |  |  |  | **ACEi group (N = 7,026)** | **ACEi group (N = 7,355)** | **ARB group (N = 48,933)** |
| Renal impairment | 6.6 | 6.1 | 0.02 | 6.5 | 6.5 | 0 |
| Rheumatoid arthritis | 0.5 | 0.3 | 0.03 | 0.5 | 0.5 | 0 |
| Schizophrenia | 0.2 | 0.1 | 0.03 | 0.2 | 0.1 | 0.04 |
| Urinary tract infectious disease | 1.4 | 0.8 | 0.05 | 1.3 | 1.4 | -0.01 |
| Viral hepatitis C | 0.3 | 0.2 | 0.02 | 0.3 | 0.2 | 0.02 |
| Visual system disorder | 8.9 | 7.9 | 0.04 | 8.9 | 9.5 | -0.02 |
| Medical history: Cardiovascular disease | | |  |  |  |  |
| Atrial fibrillation | 2.6 | 1.8 | 0.06 | 2.7 | 2.6 | 0 |
| Cerebrovascular disease | 3.8 | 5 | -0.06 | 3.9 | 4 | -0.01 |
| Coronary arteriosclerosis | 23.5 | 8.7 | 0.41 | 21.7 | 22.5 | -0.02 |
| Heart disease | 54.3 | 23.8 | 0.66 | 52.2 | 55.1 | -0.06 |
| Heart failure | 13 | 2.8 | 0.38 | 11.8 | 12.2 | -0.01 |
| Ischemic heart disease | 34.1 | 12.8 | 0.52 | 32 | 33.3 | -0.03 |
| Peripheral vascular disease | 5.9 | 5.9 | 0 | 5.9 | 6.2 | -0.01 |
| Medical history: Neoplasms | | |  |  |  |  |
| Hematologic neoplasm | 2.6 | 2.2 | 0.02 | 2.2 | 2.1 | 0.01 |
| Malignant neoplasm of anorectum | 0.8 | 0.7 | 0.01 | 0.7 | 0.6 | 0.01 |
| Malignant neoplastic disease | 9.7 | 10.6 | -0.03 | 8.7 | 8.3 | 0.01 |
| Malignant tumor of breast | 0.6 | 0.8 | -0.03 | 0.6 | 0.5 | 0.01 |
| Malignant tumor of colon | 0.9 | 1 | -0.02 | 0.9 | 0.9 | 0 |
| Malignant tumor of urinary bladder | 0.3 | 0.4 | -0.02 | 0.3 | 0.3 | -0.01 |
| Primary malignant neoplasm of prostate | 0.6 | 0.6 | -0.01 | 0.5 | 0.6 | -0.01 |

Data are presented as %.

ACEi, Angiotensin converting enzyme inhibitoir; ARB, Angiotensin receptor blocker; SMD, standardized mean difference.

**Supplementary Table 2.** Baseline characteristics of DCMC database.

|  | **ACEi group (N = 7,011)** | **ARB group (N = 19,226)** | **SMD** | **Propensity-score matched population** | | |
| --- | --- | --- | --- | --- | --- | --- |
|  |  |  |  | **ACEi group (N = 6,380)** | **ARB group (N = 6,380)** | **SMD** |
| Age group |  |  |  |  |  |  |
| 20-24 | 0.5 | 0.3 | 0.04 | 0.6 | 0.3 | 0.04 |
| 25-29 | 0.5 | 0.4 | 0.01 | 0.5 | 0.5 | 0 |
| 30-34 | 0.8 | 0.7 | 0.01 | 0.9 | 0.6 | 0.03 |
| 35-39 | 1.8 | 1.8 | 0 | 1.8 | 1.8 | 0 |
| 40-44 | 3.3 | 3.2 | 0 | 3.1 | 2.9 | 0.02 |
| 45-49 | 6.5 | 6.2 | 0.02 | 6.6 | 5.9 | 0.03 |
| 50-54 | 9.4 | 9.5 | 0 | 9.2 | 9.4 | -0.01 |
| 55-59 | 12.1 | 11.8 | 0.01 | 11.8 | 11.4 | 0.01 |
| 60-64 | 12.3 | 12.9 | -0.02 | 12.1 | 12.6 | -0.02 |
| 65-69 | 14.2 | 14.2 | 0 | 14.1 | 14.3 | 0 |
| 70-74 | 13.1 | 15 | -0.05 | 13.3 | 14.7 | -0.04 |
| 75-79 | 12.7 | 13.3 | -0.02 | 13 | 13.3 | -0.01 |
| 80-84 | 8.2 | 7.3 | 0.03 | 8.4 | 8.1 | 0.01 |
| 85-89 | 3.7 | 2.6 | 0.06 | 3.6 | 3.5 | 0 |
| 90-94 | 0.8 | 0.6 | 0.03 | 0.9 | 0.7 | 0.02 |
| 95-99 | 0.1 | 0.1 | 0.01 | 0.1 | 0.1 | 0 |
| Female | 42.7 | 52.2 | -0.19 | 45.2 | 45.9 | -0.01 |
| Medical history: General | |  |  |  |  |  |
| Acute respiratory disease | 4.4 | 3.9 | 0.03 | 4.5 | 4.4 | 0 |
| Chronic liver disease | 2.2 | 2.2 | 0 | 2.3 | 2 | 0.02 |
| Chronic obstructive lung disease | 3.9 | 3 | 0.05 | 3.9 | 3.9 | 0 |
| Dementia | 2.3 | 4.2 | -0.11 | 2.4 | 2 | 0.03 |
| Depressive disorder | 2.2 | 4.1 | -0.11 | 2.4 | 1.9 | 0.03 |
| Diabetes mellitus | 22.3 | 23.9 | -0.04 | 22.4 | 22.1 | 0.01 |
| Gastroesophageal reflux disease | 6.1 | 7.3 | -0.04 | 6 | 6.2 | -0.01 |
| Gastrointestinal hemorrhage | 3 | 2.2 | 0.05 | 2.8 | 2.9 | 0 |
| Hyperlipidemia | 22.1 | 30.3 | -0.19 | 22.8 | 21.5 | 0.03 |
| Lesion of liver | 2.8 | 2.9 | -0.01 | 2.9 | 2.8 | 0.01 |
| Obesity | 0.1 | 0.4 | -0.06 | 0.1 | 0.2 | -0.02 |
| Osteoarthritis | 1.8 | 3.3 | -0.09 | 2 | 1.7 | 0.02 |
| Pneumonia | 4.5 | 3.7 | 0.04 | 4.6 | 4.5 | 0 |
|  | **ACEi group (N = 7,011)** | **ARB group (N = 19,226)** | **SMD** | **Propensity-score matched population** | | |
|  |  |  |  | **ACEi group (N = 6,380)** | **ARB group (N = 6,380)** | **SMD** |
| Renal impairment | 5.5 | 5.4 | 0 | 5.8 | 5.6 | 0.01 |
| Rheumatoid arthritis | 1.9 | 2.5 | -0.04 | 1.9 | 1.6 | 0.02 |
| Schizophrenia | 0.2 | 0.2 | 0 | 0.2 | 0.2 | 0.01 |
| Ulcerative colitis | 0.1 | 0.1 | 0 | 0.1 | <0.1 | 0.01 |
| Urinary tract infectious disease | 2 | 1.5 | 0.04 | 2 | 1.8 | 0.01 |
| Viral hepatitis C | 0.1 | 0.3 | -0.04 | 0.2 | 0.2 | -0.02 |
| Visual system disorder | 7.5 | 10.7 | -0.11 | 7.9 | 7.8 | 0 |
| Medical history: Cardiovascular disease | | |  |  |  |  |
| Atrial fibrillation | 7.8 | 6.2 | 0.06 | 8.2 | 9.2 | -0.04 |
| Cerebrovascular disease | 5.6 | 15.6 | -0.33 | 5.9 | 4.7 | 0.05 |
| Coronary arteriosclerosis | 2.9 | 1.6 | 0.09 | 2.9 | 3.1 | -0.01 |
| Heart disease | 67.1 | 36.4 | 0.65 | 64 | 65.7 | -0.04 |
| Heart failure | 17.2 | 12.1 | 0.15 | 17.7 | 18.3 | -0.01 |
| Ischemic heart disease | 49.1 | 19.2 | 0.66 | 44.5 | 44.8 | -0.01 |
| Peripheral vascular disease | 8 | 17.1 | -0.28 | 8.5 | 7.8 | 0.03 |
| Pulmonary embolism | 1 | 0.7 | 0.03 | 1 | 0.8 | 0.02 |
| Venous thrombosis | 0.5 | 0.5 | 0.01 | 0.5 | 0.5 | 0 |
| Medical history: Neoplasms | | |  |  |  |  |
| Hematologic neoplasm | 0.5 | 0.4 | 0.01 | 0.5 | 0.3 | 0.04 |
| Malignant lymphoma | 0.2 | 0.2 | 0 | 0.2 | 0.1 | 0.02 |
| Malignant neoplasm of anorectum | 0.3 | 0.2 | 0.02 | 0.3 | 0.2 | 0.02 |
| Malignant neoplastic disease | 6 | 6.2 | -0.01 | 6 | 5.6 | 0.02 |
| Malignant tumor of breast | 0.2 | 0.3 | -0.02 | 0.2 | 0.3 | -0.02 |
| Malignant tumor of lung | 0.3 | 0.3 | 0 | 0.2 | 0.2 | 0 |
| Malignant tumor of urinary bladder | 0.2 | 0.2 | -0.01 | 0.1 | 0.3 | -0.03 |
| Primary malignant neoplasm of prostate | 0.6 | 0.5 | 0.01 | 0.5 | 0.6 | -0.01 |

Data are presented as %.

ACEi, Angiotensin converting enzyme inhibitoir; ARB, Angiotensin receptor blocker; SMD, standardized mean difference.

**Supplementary Table 3.** Baseline characteristics of KNUH database.

|  | **ACEi group (N = 1,346)** | **ARB group (N = 19,120)** | **SMD** | **Propensity-score matched population** | | |
| --- | --- | --- | --- | --- | --- | --- |
|  |  |  |  | **ACEi group (N = 1,335)** | **ARB group (N = 1,335)** | **SMD** |
| Age group |  |  |  |  |  |  |
| 20-24 | 1 | 0.3 | 0.09 | 1 | 0.7 | 0.03 |
| 25-29 | 1.6 | 0.4 | 0.12 | 1.6 | 1.8 | -0.02 |
| 30-34 | 1.7 | 0.9 | 0.08 | 1.7 | 1 | 0.06 |
| 35-39 | 3.3 | 1.8 | 0.09 | 3.2 | 3.1 | 0 |
| 40-44 | 4.2 | 3.3 | 0.04 | 4.2 | 3.4 | 0.04 |
| 45-49 | 5.6 | 5.4 | 0.01 | 5.5 | 4.9 | 0.03 |
| 50-54 | 8.1 | 8 | 0 | 8.2 | 7 | 0.04 |
| 55-59 | 11.6 | 11 | 0.02 | 11.6 | 12.2 | -0.02 |
| 60-64 | 10.6 | 11.6 | -0.03 | 10.6 | 11.8 | -0.04 |
| 65-69 | 12.2 | 13.4 | -0.04 | 12.1 | 13.4 | -0.04 |
| 70-74 | 12.8 | 14.8 | -0.06 | 12.9 | 13.5 | -0.02 |
| 75-79 | 11.4 | 13.7 | -0.07 | 11.5 | 12.1 | -0.02 |
| 80-84 | 8.6 | 9.6 | -0.04 | 8.5 | 7.9 | 0.02 |
| 85-89 | 5.6 | 4.2 | 0.07 | 5.6 | 5.5 | 0.01 |
| 90-94 | 1.4 | 1.3 | 0.01 | 1.4 | 1.3 | 0.01 |
| 95-99 | 0.4 | 0.3 | 0.03 | 0.4 | 0.4 | 0.01 |
| Female | 39.3 | 48.7 | -0.19 | 39.3 | 40.1 | -0.02 |
| Medical history: General | |  |  |  |  |  |
| Acute respiratory disease | 2.6 | 2.2 | 0.02 | 2.6 | 3.1 | -0.03 |
| Chronic liver disease | 1.3 | 0.7 | 0.07 | 1.3 | 0.7 | 0.07 |
| Chronic obstructive lung disease | 1.9 | 1.8 | 0.01 | 1.9 | 1.6 | 0.02 |
| Dementia | 2.3 | 3.2 | -0.05 | 2.3 | 1.8 | 0.04 |
| Depressive disorder | 1.7 | 3.1 | -0.09 | 1.6 | 1.9 | -0.02 |
| Diabetes mellitus | 17.9 | 7.6 | 0.31 | 17.8 | 17.7 | 0 |
| Gastroesophageal reflux disease | 2.4 | 2.9 | -0.03 | 2.4 | 2.6 | -0.01 |
| Gastrointestinal hemorrhage | 0.7 | 1.2 | -0.05 | 0.7 | 0.5 | 0.02 |
| Hyperlipidemia | 14.5 | 15.7 | -0.03 | 14.6 | 13.1 | 0.04 |
| Lesion of liver | 2.1 | 1.5 | 0.04 | 2.1 | 1.7 | 0.03 |
| Obesity | <0.4 | 0.2 | 0.02 | <0.4 | <0.4 | 0.03 |
| Osteoarthritis | 1.1 | 1.3 | -0.01 | 1.1 | 0.7 | 0.05 |
| Pneumonia | 4 | 3 | 0.06 | 3.9 | 4 | 0 |
|  | **ACEi group (N = 1,346)** | **ARB group (N = 19,120)** | **SMD** | **Propensity-score matched population** | | |
|  |  |  |  | **ACEi group (N = 1,335)** | **ARB group (N = 1,335)** | **SMD** |
| Renal impairment | 7.7 | 3.1 | 0.21 | 7.5 | 7.9 | -0.01 |
| Schizophrenia | <0.4 | 0.2 | 0 | <0.4 | 0.4 | -0.04 |
| Urinary tract infectious disease | 2.1 | 1.3 | 0.07 | 2.2 | 1.6 | 0.04 |
| Visual system disorder | 13.4 | 9.4 | 0.13 | 13.4 | 12.7 | 0.02 |
| Medical history: Cardiovascular disease | | |  |  |  |  |
| Atrial fibrillation | 2.8 | 3.4 | -0.04 | 2.8 | 2.7 | 0 |
| Cerebrovascular disease | 2.8 | 7 | -0.2 | 2.8 | 2.9 | 0 |
| Coronary arteriosclerosis | 2.9 | 2 | 0.06 | 2.9 | 3 | 0 |
| Heart disease | 34.9 | 32.9 | 0.04 | 34.8 | 37.5 | -0.06 |
| Heart failure | 8.7 | 3.4 | 0.22 | 8.5 | 8.5 | 0 |
| Ischemic heart disease | 19.1 | 11 | 0.23 | 19 | 21 | -0.05 |
| Peripheral vascular disease | 5 | 5.9 | -0.04 | 5 | 4.2 | 0.04 |
| Pulmonary embolism | <0.4 | 0.2 | 0 | <0.4 | <0.4 | 0.04 |
| Venous thrombosis | 0.4 | 0.3 | 0.03 | 0.4 | <0.4 | 0.07 |
| Medical history: Neoplasms | | |  |  |  |  |
| Hematologic neoplasm | 0.4 | 0.2 | 0.04 | 0.4 | <0.4 | 0.07 |
| Malignant neoplasm of anorectum | 1 | 0.4 | 0.07 | 1 | 0.9 | 0.01 |
| Malignant neoplastic disease | 5.1 | 4.2 | 0.04 | 5 | 4.6 | 0.02 |
| Malignant tumor of breast | <0.4 | 0.3 | -0.03 | <0.4 | <0.4 | 0.02 |
| Malignant tumor of colon | 0.6 | 0.5 | 0.01 | 0.6 | 0.7 | -0.02 |
| Malignant tumor of urinary bladder | <0.4 | 0.2 | -0.01 | <0.4 | <0.4 | -0.03 |
| Primary malignant neoplasm of prostate | 0.5 | 0.3 | 0.03 | 0.5 | 0.4 | 0.02 |

Data are presented as %.

ACEi, Angiotensin converting enzyme inhibitoir; ARB, Angiotensin receptor blocker; SMD, standardized mean difference.

**Supplementary Table 4.** Baseline characteristics of KDH database.

|  | **ACEi group (N = 5,335)** | **ARB group (N = 19,583)** | **SMD** | **Propensity-score matched population** | | |
| --- | --- | --- | --- | --- | --- | --- |
|  |  |  |  | **ACEi group (N = 4,457)** | **ARB group (N = 4,457)** | **SMD** |
| Age group |  |  |  |  |  |  |
| 25-29 | 0.7 | 0.6 | 0 | 0.7 | 0.6 | 0.02 |
| 30-34 | 0.7 | 1.4 | -0.07 | 0.8 | 0.7 | 0.01 |
| 35-39 | 2.1 | 2.8 | -0.04 | 2.2 | 1.9 | 0.02 |
| 40-44 | 3.5 | 4.4 | -0.05 | 3.5 | 2.6 | 0.05 |
| 45-49 | 7.8 | 7.6 | 0.01 | 7.5 | 7.2 | 0.01 |
| 50-54 | 9.5 | 11.4 | -0.06 | 9.3 | 8.6 | 0.02 |
| 55-59 | 12.2 | 13.5 | -0.04 | 12.4 | 11.8 | 0.02 |
| 60-64 | 12.6 | 14.3 | -0.05 | 12.8 | 13.6 | -0.02 |
| 65-69 | 12.4 | 12.4 | 0 | 12.9 | 13.6 | -0.02 |
| 70-74 | 12.3 | 11.6 | 0.02 | 12.8 | 12.9 | 0 |
| 75-79 | 10.9 | 9.2 | 0.06 | 10.8 | 11.5 | -0.02 |
| 80-84 | 7.8 | 6.3 | 0.06 | 7.7 | 7.8 | 0 |
| 85-89 | 4.9 | 2.8 | 0.11 | 4.5 | 5.2 | -0.04 |
| 90-94 | 1.8 | 1 | 0.07 | 1.6 | 1.5 | 0.01 |
| 95-99 | 0.4 | 0.1 | 0.04 | 0.3 | 0.2 | 0.01 |
| Gender: female | 41.5 | 50 | -0.17 | 44.2 | 46.1 | -0.04 |
| Medical history: General | |  |  |  |  |  |
| Acute respiratory disease | 2.6 | 2.5 | 0 | 2.6 | 2.3 | 0.02 |
| Chronic liver disease | 1.6 | 1 | 0.05 | 1.6 | 1.6 | 0 |
| Chronic obstructive lung disease | 2 | 1.3 | 0.06 | 1.9 | 1.8 | 0.01 |
| Dementia | 2.3 | 2.6 | -0.02 | 2.3 | 2 | 0.02 |
| Depressive disorder | 1.6 | 2.1 | -0.03 | 1.7 | 1.7 | 0 |
| Diabetes mellitus | 16.8 | 15.1 | 0.05 | 17.6 | 18.8 | -0.03 |
| Gastroesophageal reflux disease | 2.2 | 2.8 | -0.04 | 2.3 | 1.8 | 0.03 |
| Gastrointestinal hemorrhage | 1.5 | 1 | 0.04 | 1.4 | 1.4 | 0 |
| Human immunodeficiency virus infection | <0.1 | 0.1 | -0.01 | <0.1 | <0.1 | -0.01 |
| Hyperlipidemia | 5.9 | 8.8 | -0.11 | 6.4 | 6.6 | -0.01 |
| Lesion of liver | 3.9 | 3 | 0.05 | 3.9 | 3.7 | 0.01 |
| Obesity | 0.1 | 0.1 | -0.01 | 0.1 | <0.1 | 0.02 |
| Osteoarthritis | 0.7 | 1.6 | -0.09 | 0.7 | 0.7 | 0.01 |
|  | **ACEi group (N = 5,335)** | **ARB group (N = 19,583)** | **SMD** | **Propensity-score matched population** | | |
|  |  |  |  | **ACEi group (N = 4,457)** | **ARB group (N = 4,457)** | **SMD** |
| Pneumonia | 3.7 | 2.7 | 0.06 | 3.6 | 3.7 | 0 |
| Psoriasis | 0.2 | 0.2 | -0.02 | 0.2 | 0.1 | 0.02 |
| Renal impairment | 2.8 | 5 | -0.12 | 3 | 3 | 0 |
| Rheumatoid arthritis | 0.5 | 0.3 | 0.02 | 0.5 | 0.5 | 0 |
| Urinary tract infectious disease | 1 | 0.9 | 0.01 | 1.1 | 1 | 0.02 |
| Visual system disorder | 8.8 | 10 | -0.04 | 9.3 | 10 | -0.02 |
| Medical history: Cardiovascular disease | | |  |  |  |  |
| Atrial fibrillation | 2.6 | 1.3 | 0.1 | 2.3 | 2.9 | -0.04 |
| Cerebrovascular disease | 3.6 | 7.7 | -0.18 | 4 | 3.4 | 0.03 |
| Coronary arteriosclerosis | 0.7 | 0.3 | 0.06 | 0.7 | 0.8 | -0.02 |
| Heart disease | 55.1 | 20.5 | 0.76 | 46.4 | 47.7 | -0.03 |
| Heart failure | 14.9 | 3.6 | 0.4 | 11.3 | 11.3 | 0 |
| Ischemic heart disease | 34 | 8.4 | 0.66 | 26.3 | 26.4 | 0 |
| Peripheral vascular disease | 4.7 | 5.1 | -0.02 | 4.9 | 4.9 | 0 |
| Pulmonary embolism | 0.5 | 0.2 | 0.04 | 0.5 | 0.5 | -0.01 |
| Venous thrombosis | <0.1 | 0.2 | -0.04 | <0.1 | 0.1 | -0.02 |
| Medical history: Neoplasms | | |  |  |  |  |
| Malignant lymphoma | 0.2 | 0.1 | 0.03 | 0.2 | 0.1 | 0.02 |
| Malignant neoplasm of anorectum | 0.4 | 0.2 | 0.05 | 0.4 | 0.3 | 0.02 |
| Malignant neoplastic disease | 6.2 | 4.7 | 0.06 | 6.2 | 6.6 | -0.02 |
| Malignant tumor of breast | 0.4 | 0.5 | -0.02 | 0.4 | 0.4 | 0 |
| Malignant tumor of colon | 0.6 | 0.4 | 0.02 | 0.6 | 0.7 | -0.02 |
| Malignant tumor of lung | 0.1 | 0.1 | -0.01 | <0.1 | <0.1 | -0.01 |
| Malignant tumor of urinary bladder | 0.2 | 0.2 | -0.02 | 0.1 | 0.3 | -0.04 |
| Primary malignant neoplasm of prostate | 0.2 | 0.2 | 0 | 0.2 | 0.2 | 0.01 |

Data are presented as %.

ACEi, Angiotensin converting enzyme inhibitoir; ARB, Angiotensin receptor blocker; SMD, standardized mean difference.

**Supplementary Table 5.** Baseline characteristics of KHNMC database.

|  | **ACEi group (N = 736)** | **ARB group (N = 23,593)** | **SMD** | **Propensity-score matched population** | | |
| --- | --- | --- | --- | --- | --- | --- |
|  |  |  |  | **ACEi group (N = 734)** | **ARB group (N = 734)** | **SMD** |
| Age group |  |  |  |  |  |  |
| 25-29 | 0.7 | 0.7 | 0 | 0.7 | 0.8 | -0.02 |
| 30-34 | 1.3 | 1.5 | -0.01 | 1.4 | 1 | 0.04 |
| 35-39 | 1.3 | 3 | -0.11 | 1.4 | 1.6 | -0.02 |
| 40-44 | 4.4 | 5.2 | -0.03 | 4.5 | 3 | 0.08 |
| 45-49 | 6.3 | 7.5 | -0.05 | 6.4 | 6.1 | 0.01 |
| 50-54 | 9 | 10.8 | -0.06 | 9.1 | 11 | -0.06 |
| 55-59 | 11.3 | 13.3 | -0.06 | 11.3 | 14 | -0.08 |
| 60-64 | 11 | 13.3 | -0.07 | 11 | 10.8 | 0.01 |
| 65-69 | 13.4 | 12.3 | 0.03 | 13.5 | 13.1 | 0.01 |
| 70-74 | 14.1 | 11.8 | 0.07 | 14.3 | 13.4 | 0.03 |
| 75-79 | 10.7 | 10 | 0.02 | 10.5 | 11.4 | -0.03 |
| 80-84 | 10.3 | 6.1 | 0.15 | 9.8 | 8.6 | 0.04 |
| 85-89 | 3.9 | 3 | 0.05 | 4 | 4.1 | -0.01 |
| 90-94 | 1.5 | 0.9 | 0.06 | 1.5 | 0.8 | 0.06 |
| Female | 40.9 | 48.6 | -0.16 | 41.1 | 40.6 | 0.01 |
| Medical history: General | |  |  |  |  |  |
| Acute respiratory disease | 3.8 | 3 | 0.04 | 3.8 | 4.1 | -0.01 |
| Dementia | 2.8 | 2.6 | 0.02 | 2.9 | 2.6 | 0.02 |
| Depressive disorder | 0.9 | 1.7 | -0.06 | 1 | 1.8 | -0.07 |
| Diabetes mellitus | 23 | 22.5 | 0.01 | 22.5 | 24.5 | -0.05 |
| Gastroesophageal reflux disease | 2.7 | 2.8 | 0 | 2.6 | 2.9 | -0.02 |
| Gastrointestinal hemorrhage | 1.9 | 1.4 | 0.04 | 1.9 | 1.2 | 0.06 |
| Hyperlipidemia | 18.3 | 24.9 | -0.16 | 18.1 | 18.8 | -0.02 |
| Lesion of liver | 1.9 | 1.9 | 0 | 1.8 | 1 | 0.07 |
| Obesity | 0.8 | 0.9 | -0.01 | 0.8 | <0.7 | 0.05 |
| Osteoarthritis | 1.7 | 2.3 | -0.04 | 1.8 | 1.2 | 0.04 |
| Pneumonia | 6.4 | 2.9 | 0.17 | 6.4 | 5 | 0.06 |
| Psoriasis | <0.7 | 0.3 | -0.03 | <0.7 | <0.7 | -0.03 |
| Renal impairment | 6.3 | 7.5 | -0.05 | 6.1 | 6 | 0.01 |
| Urinary tract infectious disease | 1.3 | 2 | -0.05 | 1.4 | 2.3 | -0.07 |
| Visual system disorder | 8.3 | 9.6 | -0.05 | 8.4 | 9 | -0.02 |
|  |  |  |  |  |  |  |
|  | **ACEi group (N = 736)** | **ARB group (N = 23,593)** | **SMD** | **Propensity-score matched population** | | |
|  |  |  |  | **ACEi group (N = 734)** | **ACEi group (N = 736)** | **ARB group (N = 23,593)** |
| Medical history: Cardiovascular disease | | |  |  |  |  |
| Atrial fibrillation | 3 | 2.8 | 0.01 | 3 | 3.5 | -0.03 |
| Cerebrovascular disease | 7.7 | 7.8 | -0.01 | 7.8 | 6.7 | 0.04 |
| Coronary arteriosclerosis | 3.8 | 2.1 | 0.1 | 3.8 | 3.3 | 0.03 |
| Heart disease | 59.2 | 25.3 | 0.73 | 59.1 | 60.5 | -0.03 |
| Heart failure | 14 | 3 | 0.4 | 13.6 | 14.6 | -0.03 |
| Ischemic heart disease | 38.5 | 11.2 | 0.67 | 38.6 | 39.8 | -0.02 |
| Peripheral vascular disease | 2.4 | 1.9 | 0.04 | 2.3 | 3 | -0.04 |
| Pulmonary embolism | 0.7 | 0.3 | 0.06 | <0.7 | <0.7 | 0.07 |
| Venous thrombosis | <0.7 | 0.6 | -0.03 | <0.7 | <0.7 | -0.02 |
| Medical history: Neoplasms | | |  |  |  |  |
| Malignant neoplasm of anorectum | 0.8 | 0.3 | 0.06 | 0.7 | <0.7 | 0.02 |
| Malignant neoplastic disease | 6.4 | 5 | 0.06 | 5.4 | 4.9 | 0.02 |
| Malignant tumor of breast | 0.7 | 0.4 | 0.03 | 0.7 | <0.7 | 0.06 |
| Malignant tumor of urinary bladder | <0.7 | 0.2 | -0.01 | <0.7 | <0.7 | -0.05 |
| Primary malignant neoplasm of prostate | <0.7 | 0.3 | 0.02 | <0.7 | <0.7 | -0.02 |

Data are presented as %.

ACEi, Angiotensin converting enzyme inhibitoir; ARB, Angiotensin receptor blocker; SMD, standardized mean difference.

**Supplementary Table 6.** Baseline characteristics of PNUH database

|  | **ACEi group (N = 4654)** | **ARB group (N = 23,133)** | **SMD** | **Propensity-score matched population** | | |
| --- | --- | --- | --- | --- | --- | --- |
|  |  |  |  | **ACEi group (N = 4,474)** | **ARB group (N = 4,474)** | **SMD** |
| Age group |  |  |  |  |  |  |
| 20-24 | 0.6 | 0.7 | -0.01 | 0.6 | 0.6 | -0.01 |
| 25-29 | 0.7 | 0.6 | 0.01 | 0.7 | 0.7 | 0 |
| 30-34 | 1.2 | 1.1 | 0.01 | 1.2 | 1.1 | 0.01 |
| 35-39 | 1.2 | 2.1 | -0.07 | 1.3 | 1.4 | -0.02 |
| 40-44 | 2.8 | 3.5 | -0.04 | 2.9 | 3.2 | -0.02 |
| 45-49 | 4.1 | 4.6 | -0.03 | 4.2 | 4.3 | -0.01 |
| 50-54 | 7.4 | 8.2 | -0.03 | 7.2 | 7 | 0.01 |
| 55-59 | 11 | 11.9 | -0.03 | 11 | 10.8 | 0.01 |
| 60-64 | 13.5 | 15 | -0.04 | 13.5 | 13.8 | -0.01 |
| 65-69 | 15.8 | 14.7 | 0.03 | 15.6 | 14.5 | 0.03 |
| 70-74 | 16.4 | 15.8 | 0.02 | 16.4 | 16.8 | -0.01 |
| 75-79 | 14.5 | 12.6 | 0.05 | 14.3 | 14.2 | 0 |
| 80-84 | 7.5 | 6.8 | 0.03 | 7.5 | 8.4 | -0.03 |
| 85-89 | 2.8 | 2 | 0.05 | 2.9 | 2.6 | 0.02 |
| 90-94 | 0.5 | 0.4 | 0.02 | 0.5 | 0.6 | 0 |
| Female | 38.1 | 48.3 | -0.21 | 39.3 | 40.2 | -0.02 |
| Medical history: General | |  |  |  |  |  |
| Acute respiratory disease | 1.9 | 1.6 | 0.02 | 1.9 | 1.7 | 0.01 |
| Chronic liver disease | 2 | 3.2 | -0.08 | 2.1 | 1.4 | 0.05 |
| Chronic obstructive lung disease | 7.4 | 6.7 | 0.03 | 6.9 | 6.8 | 0 |
| Dementia | 1.6 | 2.2 | -0.04 | 1.7 | 1.6 | 0 |
| Depressive disorder | 3.2 | 4.4 | -0.06 | 3.2 | 3.1 | 0 |
| Diabetes mellitus | 22.3 | 26 | -0.09 | 22.1 | 20.3 | 0.04 |
| Gastroesophageal reflux disease | 4 | 4.6 | -0.03 | 4 | 3.6 | 0.02 |
| Gastrointestinal hemorrhage | 1.7 | 1.6 | 0.01 | 1.7 | 1.5 | 0.02 |
| Human immunodeficiency virus infection | 0.2 | 0.4 | -0.03 | 0.2 | 0.1 | 0.02 |
| Hyperlipidemia | 25.1 | 20.1 | 0.12 | 23.8 | 22.2 | 0.04 |
| Lesion of liver | 2.3 | 3.7 | -0.08 | 2.3 | 1.7 | 0.04 |
| Obesity | <0.1 | 0.4 | -0.08 | <0.1 | <0.1 | -0.03 |
| Osteoarthritis | 0.9 | 1.3 | -0.04 | 1 | 1 | 0 |
| Pneumonia | 4.4 | 4 | 0.02 | 4.2 | 4.5 | -0.02 |
|  | **ACEi group (N = 4654)** | **ARB group (N = 23,133)** | **SMD** | **Propensity-score matched population** | | |
|  |  |  |  | **ACEi group (N = 4,474)** | **ACEi group (N = 4654)** | **ARB group (N = 23,133)** |
| Psoriasis | 0.4 | 0.3 | 0.01 | 0.4 | 0.3 | 0.02 |
| Renal impairment | 7.9 | 12.3 | -0.15 | 8 | 7.9 | 0.01 |
| Rheumatoid arthritis | 0.4 | 0.7 | -0.03 | 0.4 | 0.2 | 0.03 |
| Schizophrenia | 0.1 | 0.1 | 0 | 0.1 | <0.1 | 0.04 |
| Ulcerative colitis | 0.1 | 0.1 | 0 | 0.1 | <0.1 | 0.02 |
| Urinary tract infectious disease | 1.2 | 1.7 | -0.04 | 1.3 | 1.4 | -0.01 |
| Viral hepatitis C | 1 | 1.6 | -0.06 | 1 | 0.5 | 0.05 |
| Visual system disorder | 7.2 | 11.4 | -0.14 | 7.2 | 6.5 | 0.03 |
| Medical history: Cardiovascular disease | | |  |  |  |  |
| Atrial fibrillation | 7.8 | 4.4 | 0.14 | 7.9 | 8.2 | -0.01 |
| Coronary arteriosclerosis | 10.5 | 2.8 | 0.32 | 10.2 | 9.8 | 0.01 |
| Heart disease | 73.4 | 33.4 | 0.88 | 72.4 | 73.4 | -0.02 |
| Heart failure | 16.3 | 6.2 | 0.32 | 15.3 | 15.2 | 0 |
| Ischemic heart disease | 51 | 16.1 | 0.8 | 49.3 | 49.8 | -0.01 |
| Peripheral vascular disease | 0.8 | 1.2 | -0.04 | 0.8 | 0.8 | 0 |
| Pulmonary embolism | 0.5 | 0.8 | -0.04 | 0.5 | 0.7 | -0.02 |
| Venous thrombosis | 0.8 | 1.1 | -0.03 | 0.8 | 0.8 | 0 |
| Medical history: Neoplasms | | |  |  |  |  |
| Hematologic neoplasm | 2.4 | 2.4 | 0 | 2.2 | 2.1 | 0.01 |
| Malignant lymphoma | 0.4 | 0.5 | -0.01 | 0.5 | 0.6 | -0.02 |
| Malignant neoplasm of anorectum | 0.6 | 0.5 | 0 | 0.6 | 0.4 | 0.02 |
| Malignant neoplastic disease | 11.4 | 14.4 | -0.09 | 10.7 | 9.7 | 0.03 |
| Malignant tumor of breast | 1.3 | 1 | 0.02 | 1.3 | 1 | 0.02 |
| Malignant tumor of colon | 0.7 | 0.9 | -0.03 | 0.7 | 0.8 | -0.02 |
| Malignant tumor of lung | 0.9 | 1.5 | -0.05 | 0.3 | 0.1 | 0.04 |
| Primary malignant neoplasm of prostate | 1 | 0.9 | 0.01 | 1 | 1 | -0.01 |

Data are presented as %.

ACEi, Angiotensin converting enzyme inhibitoir; ARB, Angiotensin receptor blocker; SMD, standardized mean difference.

**Supplementary Table 7.** Baseline characteristics of WKUH database.

|  | **ACEi group (N = 6,921)** | **ARB group (N = 22,593)** | **SMD** | **Propensity-score matched population** | | |
| --- | --- | --- | --- | --- | --- | --- |
|  |  |  |  | **ACEi group (N = 6,039)** | **ARB group (N = 6,309)** | **SMD** |
| Age group | |  |  |  |  |  |
| 20-24 | 0.8 | 0.7 | 0.01 | 0.8 | 0.9 | 0 |
| 25-29 | 0.6 | 0.6 | 0 | 0.6 | 0.5 | 0.01 |
| 30-34 | 0.9 | 1.2 | -0.02 | 1 | 1 | 0 |
| 35-39 | 1.9 | 1.9 | 0 | 2 | 2 | 0 |
| 40-44 | 3.4 | 3.7 | -0.02 | 3.7 | 3.6 | 0 |
| 45-49 | 6.1 | 6.2 | 0 | 6.2 | 6.5 | -0.01 |
| 50-54 | 9.3 | 9.5 | -0.01 | 9.5 | 9.7 | -0.01 |
| 55-59 | 11 | 11.5 | -0.02 | 11.1 | 11.3 | -0.01 |
| 60-64 | 13.3 | 12.8 | 0.02 | 12.9 | 13.2 | -0.01 |
| 65-69 | 15.2 | 14.1 | 0.03 | 14.9 | 15.4 | -0.01 |
| 70-74 | 14.5 | 14.3 | 0 | 14.3 | 14.2 | 0 |
| 75-79 | 11.7 | 12.7 | -0.03 | 11.7 | 11 | 0.02 |
| 80-84 | 7.3 | 7.4 | 0 | 7.4 | 7.1 | 0.01 |
| 85-89 | 3.1 | 2.9 | 0.01 | 3 | 2.9 | 0.01 |
| 95-99 | 0.2 | 0.1 | 0.03 | 0.2 | 0.1 | 0.03 |
| Gender: female | 43.9 | 48.4 | -0.09 | 45.3 | 45 | 0 |
| Medical history: General | |  |  |  |  |  |
| Acute respiratory disease | 3.4 | 2.3 | 0.07 | 2.7 | 2.8 | -0.01 |
| Chronic liver disease | 2 | 1.8 | 0.02 | 1.9 | 1.7 | 0.01 |
| Chronic obstructive lung disease | 3.2 | 2.1 | 0.07 | 2.9 | 2.8 | 0.01 |
| Dementia | 1.4 | 3.5 | -0.14 | 1.4 | 1.3 | 0.01 |
| Depressive disorder | 1.7 | 3.3 | -0.11 | 1.8 | 1.8 | 0 |
| Diabetes mellitus | 20.6 | 20.4 | 0 | 20.1 | 19.5 | 0.01 |
| Gastroesophageal reflux disease | 1.1 | 1.6 | -0.04 | 1.2 | 1.1 | 0 |
| Gastrointestinal hemorrhage | 1.8 | 1.6 | 0.01 | 1.6 | 1.6 | 0 |
| Hyperlipidemia | 18.9 | 23.5 | -0.11 | 19.4 | 17.3 | 0.05 |
| Lesion of liver | 2.7 | 2.8 | -0.01 | 2.5 | 2.5 | 0 |
| Obesity | 0.2 | 0.4 | -0.04 | 0.2 | 0.2 | 0 |
| Osteoarthritis | 1.4 | 1.7 | -0.02 | 1.5 | 1.4 | 0 |
| Pneumonia | 4.1 | 4.3 | -0.01 | 3.9 | 4.2 | -0.01 |
| Renal impairment | 5.1 | 8.1 | -0.12 | 5.1 | 4.5 | 0.03 |
|  | **ACEi group (N = 6,921)** | **ARB group (N = 22,593)** | **SMD** | **Propensity-score matched population** | | |
|  |  |  |  | **ACEi group (N = 6,039)** | **ACEi group (N = 6,921)** | **ARB group (N = 22,593)** |
| Rheumatoid arthritis | 0.5 | 1 | -0.06 | 0.6 | 0.4 | 0.02 |
| Schizophrenia | 0.2 | 0.2 | 0 | 0.2 | 0.1 | 0.01 |
| Ulcerative colitis | 0.1 | 0.1 | 0.01 | 0.1 | <0.1 | 0.03 |
| Urinary tract infectious disease | 2.2 | 1.5 | 0.05 | 2 | 2.1 | 0 |
| Viral hepatitis C | 0.1 | 0.3 | -0.04 | 0.1 | 0.1 | 0 |
| Visual system disorder | 8.5 | 8 | 0.02 | 8 | 7.5 | 0.02 |
| Medical history: Cardiovascular disease | | |  |  |  |  |
| Atrial fibrillation | 2.2 | 3.8 | -0.09 | 2.6 | 2.3 | 0.02 |
| Cerebrovascular disease | 5.9 | 13 | -0.24 | 6.3 | 6 | 0.01 |
| Coronary arteriosclerosis | 0.8 | 0.4 | 0.06 | 0.8 | 1 | -0.02 |
| Heart disease | 54.3 | 26.8 | 0.58 | 50.1 | 52.3 | -0.04 |
| Heart failure | 10.6 | 4.9 | 0.21 | 8.3 | 8.5 | -0.01 |
| Ischemic heart disease | 34.2 | 12.4 | 0.53 | 31 | 32.8 | -0.04 |
| Peripheral vascular disease | 7.3 | 11.2 | -0.14 | 6.9 | 7.2 | -0.01 |
| Pulmonary embolism | 0.3 | 0.4 | -0.01 | 0.3 | 0.3 | 0.01 |
| Venous thrombosis | 0.5 | 0.5 | 0 | 0.5 | 0.4 | 0.01 |
| Medical history: Neoplasms | | |  |  |  |  |
| Hematologic neoplasm | 0.8 | 0.7 | 0.01 | 0.7 | 0.7 | 0 |
| Malignant lymphoma | 0.3 | 0.2 | 0.01 | 0.2 | 0.3 | -0.01 |
| Malignant neoplasm of anorectum | 0.5 | 0.6 | -0.02 | 0.4 | 0.5 | 0 |
| Malignant neoplastic disease | 5.8 | 7 | -0.05 | 5.3 | 4.9 | 0.02 |
| Malignant tumor of breast | 0.4 | 0.5 | -0.01 | 0.4 | 0.3 | 0.01 |
| Malignant tumor of colon | 0.5 | 0.7 | -0.02 | 0.5 | 0.4 | 0.01 |
| Malignant tumor of lung | 0.6 | 0.8 | -0.02 | 0.1 | <0.1 | 0.01 |
| Malignant tumor of urinary bladder | 0.3 | 0.3 | -0.01 | 0.2 | 0.2 | 0 |
| Primary malignant neoplasm of prostate | 0.4 | 0.5 | -0.01 | 0.4 | 0.4 | 0 |

Data are presented as %.

ACEi, Angiotensin converting enzyme inhibitoir; ARB, Angiotensin receptor blocker; SMD, standardized mean difference.

| **Supplementary Table 8.** Baseline characteristics of ACEi group and antihypertensive agent other than RAAS inhibitor. | | | | | | |
| --- | --- | --- | --- | --- | --- | --- |
|  |  |  |  | **Propensity-score matched population** | |  |
|  | **ACEi group (N = 44,410)** | **Other group (N =91,110)** | **SMD** | **ACEi group (N = 24,238)** | **Other group (N = 24,238)** | **SMD** |
| **Age group** |  |  |  |  |  |  |
| 20-24 | 1 | 1.2 | -0.02 | 0.9 | 0.7 | 0.02 |
| 40-44 | 4.6 | 5.1 | -0.02 | 4.1 | 4 | 0.01 |
| 45-49 | 7.5 | 7 | 0.02 | 6.8 | 6.3 | 0.02 |
| 50-54 | 9.7 | 9.1 | 0.02 | 9.1 | 9 | 0 |
| 55-59 | 11.9 | 10.8 | 0.03 | 11.1 | 11.2 | 0 |
| 60-64 | 12.5 | 11.7 | 0.02 | 12.4 | 12.8 | -0.01 |
| 65-69 | 13.8 | 12.1 | 0.05 | 14.3 | 14.2 | 0 |
| 70-74 | 12.9 | 12.7 | 0 | 13.9 | 14.4 | -0.02 |
| 75-79 | 10.3 | 11.3 | -0.03 | 11.1 | 11.4 | -0.01 |
| 80-84 | 6.5 | 7.1 | -0.02 | 6.8 | 7.1 | -0.01 |
| 85-89 | 3 | 3.1 | 0 | 3.1 | 3.2 | 0 |
| Gender: female | 39.4 | 51.9 | -0.25 | 47.2 | 46.4 | 0.02 |
| **Medical history: General** |  |  |  |  |  |  |
| Acute respiratory disease | 2.5 | 3.4 | -0.05 | 3 | 3 | 0 |
| Chronic liver disease | 1.4 | 3.8 | -0.15 | 1.8 | 1.7 | 0.01 |
| Dementia | 1.6 | 2.8 | -0.08 | 2.1 | 2 | 0.01 |
| Depressive disorder | 1.8 | 3.8 | -0.12 | 2.5 | 2.5 | 0 |
| Diabetes mellitus | 22.5 | 16.2 | 0.16 | 20.4 | 21.8 | -0.03 |
| Gastroesophageal reflux disease | 3.1 | 4.4 | -0.07 | 3.4 | 3.4 | 0 |
| Gastrointestinal hemorrhage | 1.5 | 2.1 | -0.05 | 1.7 | 1.8 | 0 |
| Hyperlipidemia | 14.1 | 10.6 | 0.11 | 14.9 | 15.4 | -0.01 |
| Hypertensive disorder | 39.5 | 45.3 | -0.12 | 46.6 | 48.6 | -0.04 |
| Pneumonia | 4.1 | 5.8 | -0.08 | 4.5 | 4.6 | -0.01 |
| Renal impairment | 5.5 | 8.8 | -0.13 | 6.6 | 7.3 | -0.03 |
| Urinary tract infectious disease | 1.4 | 2.1 | -0.06 | 1.8 | 1.8 | 0 |
| Viral hepatitis C | 0.2 | 0.7 | -0.07 | 0.3 | 0.2 | 0.01 |
| Visual system disorder | 9.2 | 9.6 | -0.01 | 9.9 | 10 | -0.01 |
| **Medical history: Cardiovascular disease** |  |  |  |  |  |  |
| Atrial fibrillation | 4.2 | 2 | 0.12 | 3.6 | 4.1 | -0.03 |
| Cerebrovascular disease | 3.9 | 5.9 | -0.09 | 5.9 | 6 | -0.01 |
| Coronary arteriosclerosis | 12.7 | 2.8 | 0.38 | 6.2 | 6.9 | -0.03 |
| Heart disease | 59 | 18.5 | 0.91 | 38.6 | 42.3 | -0.08 |
| Heart failure | 14.6 | 3.1 | 0.41 | 8.3 | 8.9 | -0.02 |
| Ischemic heart disease | 38.2 | 8.3 | 0.76 | 20.9 | 22.7 | -0.04 |
| Peripheral vascular disease | 5.1 | 5.2 | -0.01 | 6.3 | 6.5 | -0.01 |
| Venous thrombosis | 0.4 | 0.8 | -0.05 | 0.5 | 0.5 | 0 |

Data are presented as %.

Abbreviations: ACEi, Angiotensin converting enzyme inhibitoir; SMD, standardized mean difference

Current table showed the aggregated balance before and after matching only for limited covariates from 7 databases.

**Supplementary Table 9**. Incidence of lung cancer in the ACEi and other cohort.

|  |  |  | Lung cancer  in ACEi group* | Lung cancer  in Other group* |  |  |  | PS-stratification | |
| --- | --- | --- | --- | --- | --- | --- | --- | --- | --- |
| Hospitals | ACEi group | Other group |  |  | Adjusted HR(95% CI) | p-value |  | Adjusted HR (95% CI) | p-value |
| AUMC | 9826 | 36674 | 1.61 (1.28-2.00) | 1.38 (1.19-1.59) | 0.96 (0.74-1.26) | 0.78 |  | 0.84 (0.61-1.16) | 0.29 |
| DCMC | 9656 | 8460 | 0.47 (0.29-0.73) | 0.30 (0.14-0.55) | 1.62 (0.69-3.77) | 0.27 |  | 0.96 (0.37-2.48) | 0.93 |
| KNUH | 2009 | 8536 | 0.66 (0.26-1.33) | 0.58 (0.47-1.09) | 0.90 (0.35-2.27) | 0.82 |  | 0.99 (0.36-2.71) | 0.99 |
| KDH | 6383 | 11609 | 1.02 (0.70-1.43) | 1.18 (0.87-1.56) | 0.73 (0.45-1.17) | 0.19 |  | 0.99 (0.56-1.78) | 0.98 |
| KHNMC | 1612 | 6506 | 1.41 (0.71-2.48) | 1.49 (0.94-2.22) | 0.96 (0.44-2.11) | 0.93 |  | 1.20 (0.47-3.07) | 0.70 |
| PNUH | 4912 | 12202 | 1.58 (1.01-2.34) | 1.58 (1.14-2.14) | 0.84 (0.50-1.43) | 0.52 |  | 0.82 (0.42-1.59) | 0.55 |
| WKUH | 10012 | 7124 | 1.07 (0.83-1.35) | 1.19 (0.89-1.55) | 0.74 (0.51-1.07) | 0.11 |  | 0.86 (0.55-1.32) | 0.49 |

^*^Incidence rate are presented as per 1000 years (95% confidential interval).

Abbreviations: ACEi, Angiotension converting enzyme inhibitor; PS, propensity-score; AUMC, Ajou University Medical Center; DCMC, Daegu Catholic University Medical Center; KNUH, Kangwon National University Hospital; KDH, Kangdong Sacred Heart Hospital; KHNMC, Kyung Hee University Hospital at Gangdong; PNUH, Pusan National University Hospital; WKUH, Wonkwang University Hospital; HR, hazard ratio; CI, confidence interval.

**Supplementary Table 10.** Incidence of lung cancer in the propensity-score matched ACEi and other cohort

| Hospitals | ACEi group | Other group | Lung cancer in ACEi group* | Lung cancer in Other group* | Adjusted HR (95% CI) | p-value |
| --- | --- | --- | --- | --- | --- | --- |
| AUMC | 6465 | 6465 | 1.27 (0.92-1.69) | 1.92 (1.44-2.49) | 0.47 (0.25-0.89) | 0.02 |
| DCMC | 3863 | 3863 | 0.38 (0.15-0.77) | 0.45 (0.18-0.90) | 0.25 (0.02-2.87) | 0.27 |
| KNUH | 1583 | 1583 | 0.80 (0.32-1.63) | 1.07 (0.46-2.06) | 0.80 (0.20-3.13) | 0.75 |
| KDH | 3531 | 3531 | 1.14 (0.69-1.75) | 0.91 (0.49-1.53) | 1.17 (0.38-3.57) | 0.79 |
| KHNMC | 1114 | 1114 | 1.69 (0.77-3.15) |  |  |  |
| PNUH | 3128 | 3128 | 1.06 (0.51-1.91) | 1.62 (0.87-2.72) | 0.37 (0.09-1.49) | 0.16 |
| WKUH | 4599 | 4599 | 1.09 (0.76-1.52) | 1.13 (0.78-1.59) | 0.72 (0.35-1.49) | 0.38 |

^*^Incidence rate are presented as per 1000 years (95% confidential interval)

Abbreviations: ACEi, Angiotension converting enzyme inhibitor; PS, propensity-score; AUMC, Ajou University Medical Center; DCMC, Daegu Catholic University Medical Center; KNUH, Kangwon National University Hospital; KDH, Kangdong Sacred Heart Hospital; KHNMC, Kyung Hee University Hospital at Gangdong.

| **Supplementary Table 11.** Baseline characteristics of ARB group and antihypertensive agent other than RAAS inhibitor. | | | | | | |
| --- | --- | --- | --- | --- | --- | --- |
|  |  |  |  | **Propensity-score matched population** | |  |
|  | **ARB group (N = 151,018)** | **Other group (N = 54,588)** | **SMD** | **ARB group (N = 30,063)** | **Other group (N = 30,063)** | **SMD** |
| **Age group** |  |  |  |  |  |  |
| **20-24** | 0.7 | 1.7 | -0.09 | 0.6 | 0.7 | -0.01 |
| **25-29** | 0.8 | 2.5 | -0.14 | 0.9 | 0.8 | 0.01 |
| **30-34** | 1.6 | 3.3 | -0.11 | 1.4 | 1.6 | -0.01 |
| **40-44** | 5.3 | 5.6 | -0.01 | 4.3 | 4.4 | 0 |
| **50-54** | 10.8 | 9.2 | 0.06 | 9.3 | 9.2 | 0 |
| **55-59** | 12.4 | 10.6 | 0.06 | 11.4 | 11.5 | 0 |
| **60-64** | 13.2 | 11.3 | 0.06 | 12.7 | 12.7 | 0 |
| **70-74** | 12.5 | 11.9 | 0.02 | 13.5 | 13.4 | 0 |
| **85-89** | 2.1 | 2.9 | -0.05 | 3.3 | 3.2 | 0.01 |
| **90-94** | 0.5 | 0.8 | -0.04 | 0.9 | 0.9 | 0 |
| **Gender: female** | 46.7 | 52.6 | -0.12 | 53.6 | 52.4 | 0.02 |
| **Medical history: General** |  |  |  |  |  |  |
| **Acute respiratory disease** | 1.9 | 3.4 | -0.09 | 2.8 | 2.7 | 0 |
| **Chronic liver disease** | 1.3 | 4.6 | -0.2 | 2.7 | 2.4 | 0.01 |
| **Chronic obstructive lung disease** | 2.4 | 3.9 | -0.09 | 3.4 | 3.3 | 0 |
| **Depressive disorder** | 2.3 | 3.6 | -0.07 | 3.3 | 3.2 | 0 |
| **Diabetes mellitus** | 20.5 | 12.5 | 0.22 | 14.3 | 14.1 | 0 |
| **Gastrointestinal hemorrhage** | 1.2 | 2.1 | -0.07 | 1.9 | 1.8 | 0.01 |
| **Hyperlipidemia** | 14.5 | 8 | 0.21 | 10.1 | 10.4 | -0.01 |
| **Hypertensive disorder** | 53 | 36.6 | 0.33 | 45.2 | 44.9 | 0 |
| **Lesion of liver** | 2.2 | 6.8 | -0.22 | 4.3 | 4 | 0.02 |
| **Osteoarthritis** | 1.5 | 1.5 | 0 | 1.8 | 1.7 | 0 |
| **Pneumonia** | 2.6 | 5.7 | -0.16 | 4.8 | 4.5 | 0.01 |
| **Psoriasis** | 0.2 | 0.2 | 0.01 | 0.2 | 0.2 | 0 |
| **Renal impairment** | 5.4 | 6 | -0.03 | 5.8 | 5.6 | 0.01 |
| **Visual system disorder** | 8.7 | 8.2 | 0.02 | 8.2 | 8.2 | 0 |
| **Medical history: Cardiovascular disease** |  |  |  |  |  |  |
| **Atrial fibrillation** | 3.1 | 1.4 | 0.12 | 1.8 | 1.8 | 0 |
| **Cerebrovascular disease** | 7 | 4.4 | 0.11 | 5.5 | 5.6 | 0 |
| **Heart disease** | 30.8 | 15.6 | 0.37 | 20.2 | 20.1 | 0 |
| **Heart failure** | 5.7 | 2.6 | 0.16 | 3.6 | 3.4 | 0.01 |
| **Ischemic heart disease** | 16.1 | 7.7 | 0.26 | 10.4 | 10.4 | 0 |
| **Peripheral vascular disease** | 6.1 | 4.2 | 0.08 | 4.9 | 4.8 | 0 |
| **Venous thrombosis** | 0.5 | 0.7 | -0.04 | 0.6 | 0.7 | -0.01 |
| **Medical history: Neoplasms** |  |  |  |  |  |  |
| **Hematologic neoplasm** | 1.2 | 3.3 | -0.14 | 2.1 | 2.1 | 0 |
| **Malignant neoplasm of anorectum** | 0.4 | 1 | -0.07 | 0.8 | 0.8 | 0 |
| **Malignant neoplastic disease** | 7.8 | 16.2 | -0.26 | 13.1 | 12.5 | 0.02 |
| **Malignant tumor of colon** | 0.6 | 1.2 | -0.06 | 1.3 | 1.1 | 0.01 |
| **Primary malignant neoplasm of prostate** | 0.5 | 0.8 | -0.04 | 0.8 | 0.8 | 0 |

Data are presented as %.

Abbreviations: ARB, Angiotensin receptor blocker; SMD, standardized mean difference

Current table showed the aggregated balance before and after matching only for limited covariates from 7 databases.

**Supplementary Table 12**. Incidence of lung cancer in the ARB and other cohort.

|  |  |  | Lung cancer  in ARB group* | Lung cancer  in Other group* |  |  |  | PS-stratification | |
| --- | --- | --- | --- | --- | --- | --- | --- | --- | --- |
| Hospitals | ARB group | Other group |  |  | Adjusted HR(95% CI) | p-value |  | Adjusted HR (95% CI) | p-value |
| AUMC | 9826 | 36674 | 1.06 (0.91-1.23) | 1.22 (0.98-1.49) | 0.69 (0.53-0.89 | 0.004 |  | 0.75 (0.54-1.03) | 0.08 |
| DCMC | 9656 | 8460 | 0.35 (0.23-0.51) |  | 1.53 (0.44-5.32) | 0.50 |  | 2.38 (0.60-9.52) | 0.22 |
| KNUH | 14328 | 3088 | 0.36 (0.22-0.54) |  | 1.02 (0.29-3.60) | 0.97 |  | 1.40 (0.38-5.17) | 0.61 |
| KDH | 6383 | 11609 | 0.69 (0.50-0.92) | 1.43 (0.95-2.05) | 0.52 (0.32-0.85) | 0.008 |  | 0.64 (0.37-1.12) | 0.12 |
| KHNMC | 1612 | 6506 | 0.90 (0.69-1.14) | 2.60 (1.39-4.35) | 0.39 (0.21-0.73) | 0.003 |  | 0.39 (0.20-0.77) | 0.006 |
| PNUH | 18576 | 7395 | 1.14 (0.87-1.47) | 1.99 (1.35-2.80) | 0.62 (0.39-0.97) | 0.04 |  | 0.64 (0.38-1.08) | 0.09 |
| WKUH | 23763 | 5207 | 1.29 (1.08-1.53) | 1.20 (0.84-1.65) | 0.94 (0.64-1.39) | 0.77 |  | 0.989 (0.64-1.51) | 0.93 |

^*^Incidence rate are presented as per 1000 years (95% confidential interval).

Abbreviations: ARB, Angiotension receptor blocker; PS, propensity-score; AUMC, Ajou University Medical Center; DCMC, Daegu Catholic University Medical Center; KNUH, Kangwon National University Hospital; KDH, Kangdong Sacred Heart Hospital; KHNMC, Kyung Hee University Hospital at Gangdong; PNUH, Pusan National University Hospital; WKUH, Wonkwang University Hospital; HR, hazard ratio; CI, confidence interval.

**Supplementary Table 13.** Incidence of lung cancer in the propensity-score matched ARB and other cohort

| Hospitals | ARB group | Other group | Lung cancer in ARB group* | Lung cancer in Other group* | Adjusted HR (95% CI) | p-value |
| --- | --- | --- | --- | --- | --- | --- |
| AUMC | 12911 | 12911 | 0.88 (0.64-1.18) | 1.46 (0.96-1.60) | 0.89 (0.46-1.73) | 0.74 |
| DCMC | 4208 | 4208 | 0.38 (0.14-0.83) |  | 1.00 (0.12-8.33) | 0.99 |
| KNUH | 2961 | 2961 | 0.49 (0.17-1.05) |  |  |  |
| KDH | 5564 | 5564 | 0.97 (0.59-1.50) | 1.39 (0.89-2.06) | 1.00 (0.34-2.92) | 0.99 |
| KHNMC | 2493 | 2493 | 1.27 (0.58-2.36) | 2.77 (1.48-4.65) | 0.17 (0.02-1.75) | 0.13 |
| PNUH | 5936 | 5936 | 1.67 (1.08-2.45) | 1.95 (1.28-2.82) | 1.71 (0.66-4.43) | 0.27 |
| WKUH | 4299 | 4299 | 1.25 (0.81-1.80) | 1.21 (0.82-1.72) | 1.00 (0.44-2.25) | 0.99 |

^*^Incidence rate are presented as per 1000 years (95% confidential interval)

Abbreviations: ARB, Angiotension receptor blocker; PS, propensity-score; AUMC, Ajou University Medical Center; DCMC, Daegu Catholic University Medical Center; KNUH, Kangwon National University Hospital; KDH, Kangdong Sacred Heart Hospital; KHNMC, Kyung Hee University Hospital at Gangdong.
